# Vulture: cloud-enabled scalable mining of microbial reads in public scRNA-seq data

**DOI:** 10.1093/gigascience/giad117

**Published:** 2024-01-09

**Authors:** Junyi Chen, Danqing Yin, Harris Y H Wong, Xin Duan, Ken H O Yu, Joshua W K Ho

**Affiliations:** Laboratory of Data Discovery for Health Limited (D^2^4H), Hong Kong Science Park, Hong Kong SAR, China; School of Biomedical Sciences, Li Ka Shing Faculty of Medicine, The University of Hong Kong, Pokfulam, Hong Kong SAR, China; Laboratory of Data Discovery for Health Limited (D^2^4H), Hong Kong Science Park, Hong Kong SAR, China; School of Biomedical Sciences, Li Ka Shing Faculty of Medicine, The University of Hong Kong, Pokfulam, Hong Kong SAR, China; Laboratory of Data Discovery for Health Limited (D^2^4H), Hong Kong Science Park, Hong Kong SAR, China; Laboratory of Data Discovery for Health Limited (D^2^4H), Hong Kong Science Park, Hong Kong SAR, China; Laboratory of Data Discovery for Health Limited (D^2^4H), Hong Kong Science Park, Hong Kong SAR, China; School of Biomedical Sciences, Li Ka Shing Faculty of Medicine, The University of Hong Kong, Pokfulam, Hong Kong SAR, China; Laboratory of Data Discovery for Health Limited (D^2^4H), Hong Kong Science Park, Hong Kong SAR, China; School of Biomedical Sciences, Li Ka Shing Faculty of Medicine, The University of Hong Kong, Pokfulam, Hong Kong SAR, China

**Keywords:** cloud computing, single cell, COVID-19, HCC, virus

## Abstract

The rapidly growing collection of public single-cell sequencing data has become a valuable resource for molecular, cellular, and microbial discovery. Previous studies mostly overlooked detecting pathogens in human single-cell sequencing data. Moreover, existing bioinformatics tools lack the scalability to deal with big public data. We introduce Vulture, a scalable cloud-based pipeline that performs microbial calling for single-cell RNA sequencing (scRNA-seq) data, enabling meta-analysis of host–microbial studies from the public domain. In our benchmarking experiments, Vulture is 66% to 88% faster than local tools (PathogenTrack and Venus) and 41% faster than the state-of-the-art cloud-based tool Cumulus, while achieving comparable microbial read identification. In terms of the cost on cloud computing systems, Vulture also shows a cost reduction of 83% ($12 vs. ${\$}$70). We applied Vulture to 2 coronavirus disease 2019, 3 hepatocellular carcinoma (HCC), and 2 gastric cancer human patient cohorts with public sequencing reads data from scRNA-seq experiments and discovered cell type–specific enrichment of severe acute respiratory syndrome coronavirus 2, hepatitis B virus (HBV), and *Helicobacter pylori*–positive cells, respectively. In the HCC analysis, all cohorts showed hepatocyte-only enrichment of HBV, with cell subtype-associated HBV enrichment based on inferred copy number variations. In summary, Vulture presents a scalable and economical framework to mine unknown host–microbial interactions from large-scale public scRNA-seq data. Vulture is available via an open-source license at https://github.com/holab-hku/Vulture.

## Background

Pathogenic diseases are considered a significant threat to global health, such as severe acute respiratory syndrome coronavirus 2 (SARS-CoV-2) in coronavirus disease 2019 (COVID-19), hepatitis B virus (HBV) and hepatitis C virus (HCV) in hepatocellular carcinoma (HCC) [1], and *Helicobacter pylori* in gastric cancer (GC) [2]. Single-cell or single-nucleus RNA sequencing (sc/snRNA-seq) has reformed the investigation of complex diseases and contributed to discoveries of host–microbial interaction mechanisms [3–7]. Due to the rapidly maturing scRNA-seq technologies, the exponentially growing public scRNA-seq data resources have become a gold mine for conducting *in silico* investigations toward host–microbial interactions.

In the current practice of scRNA-seq data processing, a key concern is the selection of the reference genomes when quantifying the reads. Most studies only align reads to the host genome or focus on limited microbial genomes [8–12]. This practice systematically risks missing either the known or unknown host–microbial interactions in the datasets. It is therefore worthwhile to perform reanalyses of existing public scRNA-seq data on the cloud to uncover the breadth of these interactions. According to the Human Cell Atlas [13] Data Portal, as of December 2022, there are an estimated 12.3 M cells from 2,400 specimens, totaling 38.1 TB in file size of published human cellular droplet-based scRNA-seq data. As more and more files become available, cloud computing becomes increasingly enticing as the choice for performing large-scale reanalyses that can leverage huge amounts of computational resources without the need to purchase or maintain expensive hardware and avoid the transfer of large amounts of data.

Several tools have been developed for the identification of microbial reads in human scRNA-seq data on local machines. Viral-Track [14] is an existing computational pipeline that detects viral–host interactions in droplet scRNA-seq data by scanning host-unmapped reads for the presence of viral RNA. Based on a similar schema, Zhang et al. and Lee et al. developed PathogenTrack [15] and Venus [16], which have added capabilities. PathogenTrack can quantify bacteria in addition to viruses; the tool was benchmarked to be mostly correlated in microbial unique molecular identifiers (UMIs) called as and faster in runtime than Viral-Track [15]. Venus identifies viruses only but has another module to discover viral integration sites. However, due to the number of steps and certain tool choices in these pipelines, their scalability can still be improved.

These off-the-shelf microbial calling methods for scRNA-seq are developed as command-line tools running on local computing environments, which may eventually struggle with the scale of published data on the cloud. Only with cloud computing can we obtain the scRNA-seq big data as well as leverage a huge amount of computational resources without the maintenance of expensive devices. Previously, we [17] developed the cloud-based Falco framework for scalable scRNA-seq analysis. On 2 public scRNA-seq datasets, it was 2.6 to 145.4 times faster than running on the local computing environments. Li et al. [18] developed a scalable scRNA-seq analysis framework based on the Terra platform called Cumulus afterward. With Cumulus, Delorey et al. [19] performed COVID-19 scRNA-seq dataset analysis on 420 specimens from 11 organs in 2021. In 2022, Edgar et al. [20] developed the Serratus framework. They reviewed 5.7 million transcriptome sequencing (RNA-seq) data for RNA-dependent RNA polymerases and identified more than 105 novel RNA viruses. However, Falco only has supported the Smart-Seq protocol since 2017, which is insufficient nowadays. Also, neither the Cumulus nor the Serratus pipeline focus on the host–microbial scRNA-seq analysis.

To perform a large-scale meta-analysis of public scRNA-seq data, we developed Vulture, which to our knowledge is the first cloud-based scalable framework for discovering microbial reads in public scRNA-seq data. It can be executed either on the cloud container services in parallel or in a local environment. Our tool provides an easily modifiable, host–microbial combined reference that standardizes the gene transcript annotations of human and known human–host viruses and bacteria. Additional features of our Vulture are the support of multiple formats of raw sequencing file inputs and the quality control metrics of the identified intracellular microbial UMIs. We benchmarked the scalability and cost-effectiveness of our tool and show it outperforms existing solutions. With Vulture, we reanalyzed cohorts of COVID-19, hepatocellular carcinoma, and gastric cancer with public raw sequencing data of droplet scRNA-seq and examined the host–microbial interactions of SARS-CoV-2, HBV, and *H. pylori*, respectively. Specifically, we detected an upregulation of chemokine receptor crosstalk along with the coinfection of SARS-CoV-2 and human metapneumovirus (hMPV) from a COVID-19 bronchoalveolar lavage fluid (BALF) sample and potential HBV-induced copy number variations from an HCC sample. The result shows the utility of viral calling to the full set of known host microbes.

## Methods

### Cloud infrastructure of Vulture on AWS batch infrastructure with Nextflow

The cloud framework of Vulture is described in Supplementary Fig. S1a. Vulture applications on the cloud are constructed in Docker containers. A container is a lightweight software unit that packages all our procedures and dependencies for sequence alignment, quality control, and downstream analysis. Containerized Vulture applications are managed by the Amazon Web Services (AWS) Batch service. AWS Batch is a batch management capability to efficiently run a huge amount of batch computing jobs on AWS. The Batch is a job scheduler composed of 4 elements, including Compute Environments, Job queues, Job definitions, and Jobs. The Compute Environment specifies the computational resources required for a type of task. We applied the SPOT_CAPACITY_OPTIMIZED allocation strategy in Batch to prioritize the use of spot instances in Compute Environment. The Job Queue maps the Vulture pipeline task to 1 or more Compute Environments. The Job Definition is a template that assigns the Docker image to be employed in running a particular task along with its parameters such as the number of CPUs, the amount of memory, and other configurations. The Jobs binds a Job Definition to a specific Job Queue and executes the task command in the Docker container. In the Vulture pipeline, Job definitions and execution of Jobs are controlled by Nextflow, a language that streamlines the deployment of workflows on the commercial cloud and clusters. Nextflow creates the required Job Definitions and Jobs as needed. Each Job can use a different queue and Docker image. The Vulture container is published in DockerHub and Elastic Container Registry (ECR) that are accessible from the instances run by Batch. The Simple Storage Service (S3) bucket is where the input, output, and working directory of the Vulture pipeline are stored during execution.

### Construction of host–microbe combined reference genome

The first step of Vulture is to construct reference genomes and corresponding annotations for the host (human, in this study) and host–infection viruses and bacteria. We use 245 distinct human–host prokaryotes curated by the NCBI Genome [21] and 529 human–host viral species from viruSITE [22], which together with the human reference genome hg38 form a combined reference set. The set is a collation of the reference genome fasta sequences and exon/transcript/gene gtf annotations of all species used. Nonhost exons with a minimap2 (RRID:SCR_018550) [23] alignment to the host genome were removed due to ambiguity. The combined host–microbe reference genome was indexed using the ***genomeGenerate*** module from the STARsolo (RRID:SCR_021542) [24] tool.

### Quantifying reads from scRNA-seq data to count matrices

Vulture supports a variety of alignment algorithms to quantify scRNA-seq sequences with the constructed combined reference genome. Users can select STARsolo [24] (default), Cell Ranger [25], Kallisto | bustools [26], and Alevin [27]. Sequence data can be quantified as a 2-dimensional UMI count matrix of cells × genes and the corresponding Binary Sequence Alignment Map (BAM). BAM files are only generated if STARsolo or Cell Ranger is selected.

### Quality control of the mapped microbial reads and count matrix

After obtaining the results of the sequence alignment, we also perform additional quality control steps to increase the likelihood that the viral sequences found are intracellular and reliable. Vulture utilizes the EmptyDrops [28] algorithm to filter out the droplets with noncellular ambient RNA. We then optionally perform various quality analyses on the BAM files documenting the sequence alignments, including multimapping of the resulting sequences to host or nonhost genes and reads dispersion, which is the extent of unique positions the reads are aligned on a given transcript.

### Downstream analysis of scRNA-seq samples

The meta-analysis of the Vulture processed results is composed of several procedures. We applied Seurat (RRID:SCR_007322) [29] for the COVID-19, HCC, and GC samples listed in Table 1 to perform scRNA-seq processing and clustering, respectively. For the batch effects removal across different cohorts, we applied Harmony [30] for the COVID-19, HCC, and GC samples. CellChat (RRID:SCR_021946) [31] is applied to calculate the ligand–receptor interactions among the annotated cell types. Copy number variation (CNV) inference and clone identification for the HCC sample are analyzed by the inferCNV (RRID:SCR_021140) [32] package.

**Table 1:** Overview of the datasets processed in this study.

Data name	SRP accession	Disease	Tissue	No. of runs	No. of patients	Format
Liao et al. [33]	SRP250732	COVID-19	BALF	12	12	fastq
Bost et al. [14]	SRP279746	COVID-19	BALF	336	22	fastq
Losic et al. [8]	SRP136347	HCC	Tumor	7	2	bam
Sharma et al. [9]	SRP278381	HCC	Tumor	58	16	bam
Ho et al. [10]	SRP318499	HCC	Tumor	8	8	bam
Zhang et al. [11]	SRP215370	GC	Tumor	16	2	fastq
Kim et al. [12]	SRP261119	GC	Tumor	13	13	fastq

### Cell-type enrichment of microbial UMI

We follow the idea in [19] to calculate the cell type–specific enrichment score of intracellular microbes. The reason is that the number of microbial UMIs is small, and their differences across cell types are difficult to observe. The enrichment score for cluster C in the clustering of cells is computed as follows:


\begin{eqnarray*}
\textit{Enrichment}\left( C \right)\ = \ log\left( {\frac{{N_V^C\ + \ \varepsilon }}{{{N}_V \times \ {P}_C\ + \ \varepsilon }}} \right) \end{eqnarray*}

$N_V^C$ is the number of microbe-positive cells in cluster C, ${N}_V$ is the number of microbe-positive cells in the whole cohort, ${P}_C$ is the proportion of the total number of cells in cluster C out of the total number of cells in the cohort, and $\ \varepsilon$ is a small float to avoid zero subtractions.

The *P* value of cell type–specific enrichments of intracellular microbes was calculated by randomly permuting the identical number of microbes’ positive annotations to all cell types 10,000 times. The empirical *P* value is the proportion of permutations that get an enrichment score not less than the actual score in the cohort out of 10,000 times. We also perform the false discovery rate (FDR) correction on the empirical *P* value.

## Analyses

### Vulture: a cloud-based microbial calling framework for public scRNA-seq data

**Figure 1:**
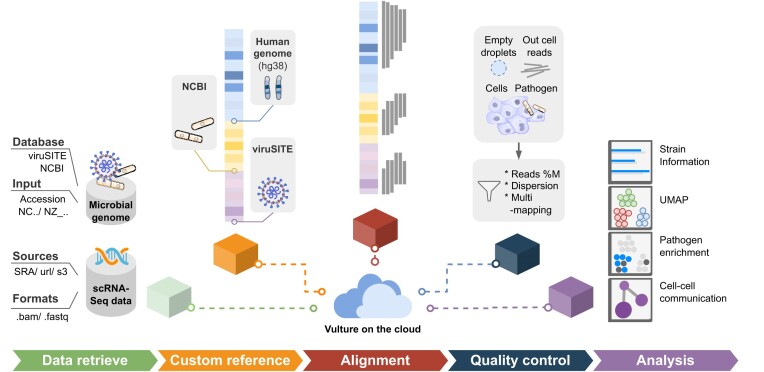
Schematic diagram of Vulture for scRNA-seq microbial calling on the cloud. Vulture is a containerized computational framework composed of 5 procedures. The 5 steps include (i) multiformat scRNA-seq data and microbial genome retrieval, (ii) custom combined reference construction, (iii) reads alignment, (iv) quality control, and (v) downstream analysis. All procedures in the Vulture architecture run as containerized applications on the Amazon Batch service.

The architecture of Vulture is shown in Fig. 1. Vulture is composed of a bioinformatics analysis container, a cloud platform, and a workflow management tool. The container defines 5 main processes for performing microbe calling for sc/snRNA-seq data: sequence data retrieval, human–microbe combined reference construction (optional), reads alignment, quality control, and downstream analysis (optional). Detailed implementations are listed in the Methods section and Supplementary Fig. S1a. Vulture receives 2 major inputs by default: (i) the sequencing files and (ii) microbe genome files. The input sequencing files can be a set of run accession numbers (prefixed by SRR) from the Sequence Read Archive (SRA) to a set of Amazon S3 or HTTP downloaded URLs. Both fastq and bam files are supported. As for the input of combined reference, we provide a default host–microbe reference covering human and all human–host microbe genomes. Users can also build their custom combined genome by inputting a list of microbe genome accession numbers based on viruSITE or NCBI.

Vulture utilizes cloud computing to provide a fast, scalable, and cost-effective viral calling framework without the need for hardware maintenance. Vulture is built on the AWS Batch service natively, which efficiently runs a huge amount of computing jobs while optimizing compute resources. At the same time, Vulture is implemented by a Docker container and can be easily run on local servers or other cloud platforms. We applied Nextflow (RRID:SCR_024135), a workflow management language to deploy complex parallel workflows of containers on clouds and clusters. The cloud architecture of Vulture is described in the Method section and Supplementary Fig. S1b. Through an AWS Batch and Nextflow, users can run thousands of viral calling tasks for public scRNA-seq data in parallel with simple configuration inputs.

### Runtime performance and cost-effectiveness of Vulture on the cloud

**Figure 2:**
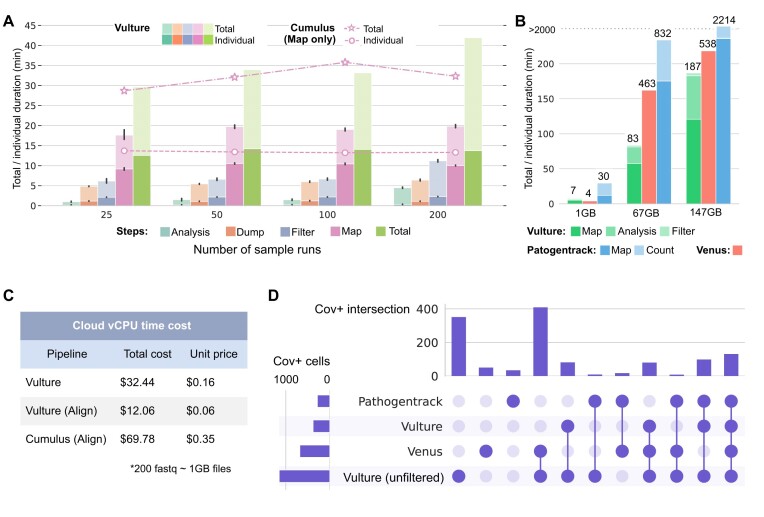
Performance benchmark of the Vulture. (A) Performances of the Vulture pipeline to run 25 to 200 parallel analyses. The performance is measured by execution duration (pastel) and vCPU time (saturated) with the respective number of parallel tasks. The time of each of the 4 steps in the Vulture pipeline is displayed separately. The line plot is the time for Cumulus to run 25 to 200 parallel read alignment tasks in comparison to the mapping step of Vulture. Total time (star-marked) and individual task durations (circle-marked) of different numbers parallel run processed by the 2 pipelines. (B) Task duration comparison among viral calling methods to run a single analysis with different input file sizes. The local version of Vulture is composed of 3 steps (Map, Analysis, and Filter), Pathogentrack is composed of 2 steps (Map and Count), and Venues is end-to-end. The total duration of tasks is presented in stacked bar plots. (C) Cost (in US dollars) of the computation resource needed to run 200 analyses on cloud platforms of Vulture and Cumulus. (D) Consistency among viral calling methods. The consistency is estimated by measuring the intersections of virus-positive cells annotated by different tools. Vulture results and results before the filtering step are discussed separately.

To validate and benchmark the scalability of Vulture on the cloud, we tested it through the public COVID-19 scRNA-seq data by Bost et al. [14] consisting of up to 400 individual fastq files. Execution duration (pastel) and vCPU time (saturated) from retrieving files to bam analysis of running 25 to 200 parallel tasks are recorded in Fig. 2A. Vulture analysis on 200 fastq files was within 48 minutes, reaching a speed (compared to running 200 single tasks sequentially) of 155×, showing that it is highly scalable. We also compared Vulture to another cloud-based tool Cumulus [18] in read mapping on an identical prebuilt host–microbe genome because Cumulus did not natively support viral calling tasks. Figure 2A indicates that Vulture outperformed Cumulus nearly 2-fold in total duration (saturated). Costing ${\$}$12, Vulture runs 200 alignment tasks in 20 minutes, while Cumulus needs ${\$}$69 to run 200 samples in 32 minutes. Vulture takes advantage of the AWS Batch spot capacity optimization technique. Its utilization of spot instances reduced the cost of running viral calling pipelines. Also, it ensured the availability of computational resource allocation, maximizing the number of concurrent tasks and minimizing the response time of pending tasks.

### Performance of Vulture on the local environment against off-the-shelf tools

We also tested Vulture local command-line tools against Venus and PathogenTrack [15, 16] using 3 COVID-19 scRNA-seq samples with different sizes: a 1-GB small sample (SRR12570205) from Bost et al. [14], a 67-GB medium sample (SRR11537951), and a 141-GB large sample (SRR11181956) from Liao et al. [33]. Fig. 2B indicated that Vulture is the most computationally effective method among the three. On medium and large samples, Vulture took 83 and 187 minutes to finish the analysis. It was 2- to 3-fold faster than Venus (463 and 538 minutes, respectively) and 9- to 10-fold faster than PathogenTrack (832 and 2,214 minutes, respectively). Besides, we tested the consistency among methods on 3 datasets by measuring the intersection of SARS-CoV-2–positive cells in Fig. 2B. The result of Vulture before filtering the empty droplets [28] named “Vulture (unfiltered)” is also added to the comparison. Since Vulture (unfiltered) is a superset of the Vulture result, the intersection between the two is the Vulture set in the figure. Fig. 2D indicates that Vulture reaches consensus with the others in most cases because most major sets are intersections with other methods. After all, the intersection of the 3 methods (which is usually the smallest) is the third largest in the test. Vulture’s result is most consistent with Venus with the largest set of intersections. Vulture (unfiltered) is the most sensitive with the largest number of SARS-CoV-2–positive cells (Fig. 2D) that cover most of the cells identified by others (Supplementary Fig. S2a). It is the quality control step that filtered out many empty droplets. On 135 cells, the intersection of the three, we calculated the mean absolute error (MAE) and Pearson correlation of SARS-CoV-2 viral UMI counts across methods in Supplementary Fig. S2c and d. The MAEs between Vulture, Venus, and PathogenTrack are smaller than 1, showing that Vulture consistently generated microbial calling results compared to state-of-the-art methods in a faster manner.

### Vulture enables cloud-based discovery of metapneumovirus reads in COVID-19 BALF samples

SARS-CoV-2 infection has been identified to be the source of the worldwide COVID-19 pandemic since 2019. Many aspects of how the viral–host interaction have remained unrevealed. There is a major interest in identifying coinfection of other pathogens in patients with COVID-19. Therefore, we applied Vulture on the cloud on BALF samples from the SRA to call viruses. We performed a meta-analysis on the Liao et al. [33] cohort from China (SRP250732) and the Bost et al. [14] cohort (SRP279746) from Israel. We ran a downstream analysis on BALF samples for 2 cohorts, totaling 51,338 and 991,722 cells after all QC filtering, respectively. After preprocessing and clustering, the cell types were defined based on marker genes from Bost et al. and Liao et al. (Supplementary Fig. S3b and c). Given the fact that SARS-CoV-2 UMIs in scRNA-seq data are relatively low and imbalanced, a statistical test (see Methods) [19] is performed to estimate cell type–specific enrichment of SAR-CoV-2 infection.

**Figure 3:**
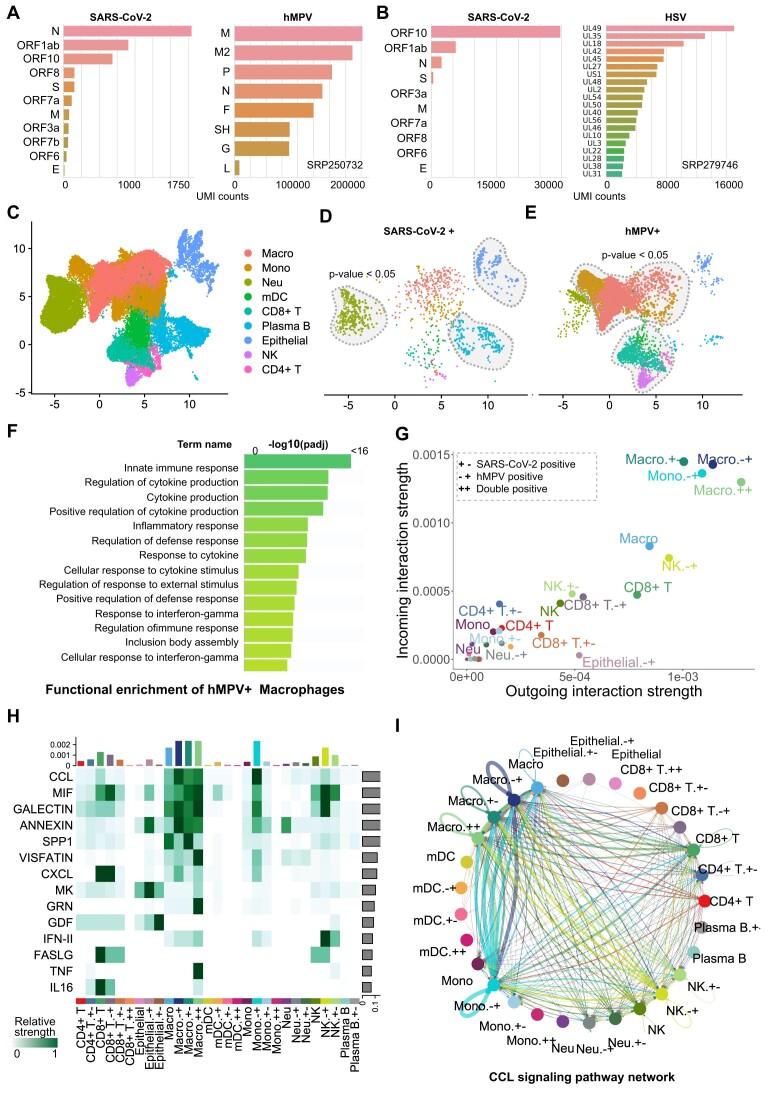
Viral calling meta-analysis results on the COVID-19 BALF samples. (A, B) Transcript UMIs of 3 major detected viruses (SARS-CoV-2, hMPV, and HSV) from SRP250732 and SRP 279746, respectively. (C) UMAP plot of the COVID-19 BALF data; cells are colored by cell-type annotations. (D, E) UMAP plots of the SARS-CoV-2 and hMPV infection, respectively. Infected cells are colored orange while other cells are gray. (F) The top 15 enriched Gene Ontology (GO) terms identified by functional enrichment analysis. (G) Cell–cell interaction (CCI) strengths across different cells grouped by cell types along with viral infections. (H) Enriched signaling pathways identified by CellChat. (I) The CCI among cell types through the CCL signaling pathway network.

The combined microbe–host genome in Vulture includes a comprehensive set of human–host microbes to identify coinfections or unaware microbes. Vulture revealed human metapneumovirus (hMPV) coinfection with SARS-CoV-2 in the Liao et al. [33] cohort (SRP250732) and unexpected herpes simplex viruses (HSV) in the Bost et al. [14] cohort (SRP279746), consistent with previous findings [14]. UMIs for different viral transcripts are in Fig. 3A and B. Cell-type visualization of BALF cells in the Liao cohort is in Fig. 3C, with SARS-CoV-2 and hMPV presence in Fig. 3D and E. UMAP plots showing the distribution of and SARS-CoV-2 and HSV for the Bost cohort are presented in Supplementary Fig. S3a. Statistical tests found SARS-CoV-2 enriched (*P* < 0.05) in epithelial cells, neutrophils, and plasma B cells (Fig. 3D and Supplementary Table S1), as well as hMPV enriched in CD8^+^ T cells, natural killer cells, macrophages, and monocytes (Fig. 3E and Supplementary Table S3). Fig. 3E shows a separate monocyte subtype with hMPV infections. Therefore, we compare the differential expressed genes between the hMPV-enriched monocytes/macrophages to the hMPV-negative monocytes/macrophages. Differentially expressed genes for the virally infected subtype are shown in Supplementary Table S7. S100A8/S100A9 were upregulated in hMPV-enriched macrophages/monocytes and involved in neutrophil-related inflammation [34]. FCN1 upregulated, encoding a complement cascade member [3]. IDO1 upregulated, and murine coronavirus infection activate dAhR independently, affecting cytokines [35]. g:Profiler [36] identified functional enrichment of the top 100 upregulated genes in hMPV-enriched subtypes. Fig. 3F results indicate subtypes’ patterns drive innate immune, cytokine responses. Interferon-gamma (IFN-γ) response activates IFN response in alveolar macrophages, recruits monocyte-derived alveolar macrophages, and forms an inflammatory signaling circuit [34].

We analyzed SARS-CoV-2 and hMPV coinfection cell–cell interactions (CCIs) using CellChat (RRID:SCR_021946) [31]. Cell types are divided into subgroups: SARS-CoV-2–positive (+–), hMPV-positive (–+), double-positive (++), and normal cells. Virus-infected macrophages and hMPV-infected monocytes showed stronger CCIs than normal cells (Fig. 3G). The Chemokine Signaling Pathways (CCL and CXCL) were significant (Fig. 3H and I) in virally infected macrophages and hMPV-infected monocytes. These pathways play a role in severe COVID-19, SARS, and Middle Eastern respiratory virus [37]. Key ligand–receptor pairs in the CCL pathway are CCL3/5/7/8 binding to CCR1/5 (Supplementary Fig. S4), driving monocyte recruitment, managing subsequent macrophage differentiation, and activating more immune cells, causing lung epithelial damage [38]. The macrophage migration inhibitory factor (MIF) pathway predicts the outcome of acute respiratory distress syndrome (ARDS) and hallmarks severe COVID-19 [39]. The GALECTIN pathway also exhibits strong interactions, influencing infection consequences [40]. ANNEXIN and SPP1 (Fig. 3H) also contribute strong CCI among monocytes and macrophages.

### Cloud-based meta-analysis reveals an HBV-associated CNV signature in HCC

Another advantage of having a cloud-based framework is that it facilitates the integration of multiple datasets that are already in the same repository on the cloud. We performed a meta-analysis of the 3 public HCC cohorts with droplet scRNA-seq sequencing data, SRP278381, SRP136347, and SRP318499. We ran Vulture on the HCC samples of 24 patients in these cohorts, totaling 421,780 cells following all QC filtering. After clustering and integration, the cell types were defined based on marker genes from Sharma et al. [40] (SRP278381) (Supplementary Fig. S5c). Microbial enrichment detection (Supplementary Table S4) on each of the cohort’s indicated hepatocytes is the only cell type with HBV enrichment (Supplementary Fig. S5b).

**Figure 4:**
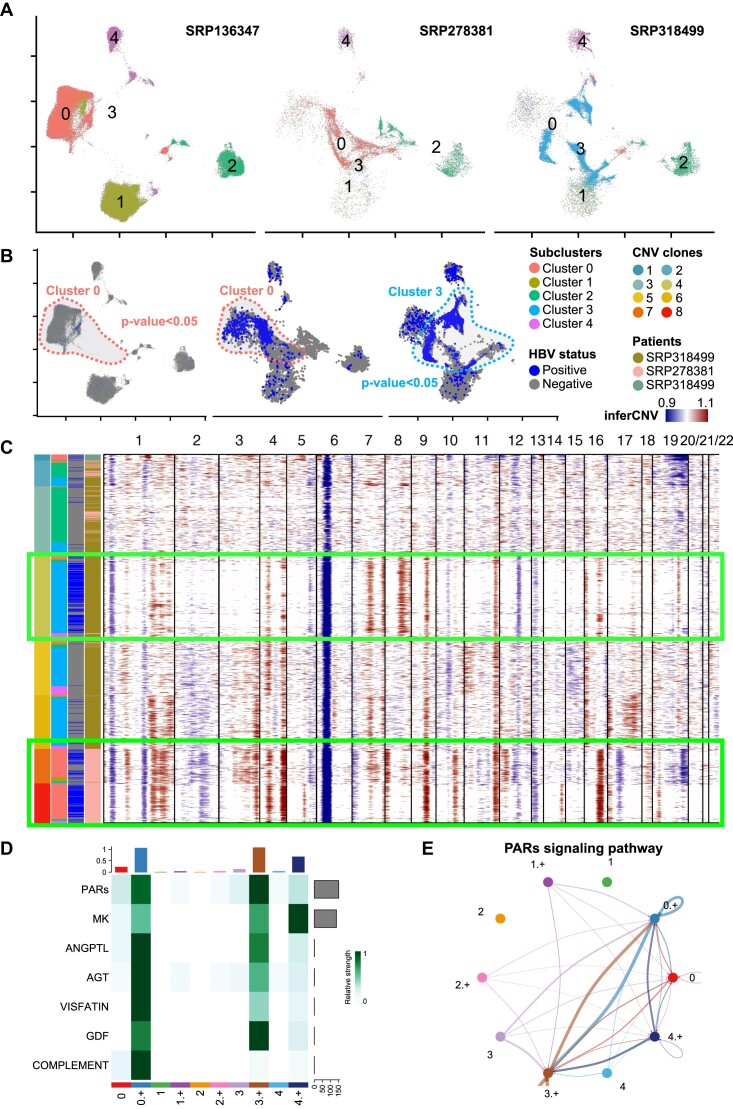
Viral calling meta-analysis results on the HCC hepatocytes. (A) UMAP plot of the HBV-enriched hepatocytes; cells are colored by subclustering annotations. (B) UMAP plots of the intracellular HBV. HBV-positive cells are colored blue while other cells are gray. (C) The top 15 enriched Gene Ontology (GO) terms identified by functional enrichment analysis. (C) Heatmap of CNV inferred by inferCNV across chromosomes on the HCC hepatocytes. Cells are labeled by different annotations including CNV clones, subclusters, HBV status, and patients. (D) Enriched signaling pathways identified by CellChat of hepatocytes. (E) The CCI among cell types through the PAR signaling pathway network.

To further delineate the HBV enrichment within hepatocytes, we reclustered and reintegrated the hepatocytes of 11 of 24 patient samples that contained any HBV expression (Fig. 4A) and found that the only HBV-enriched subclusters were 0 and 3 (Supplementary Table S5), with both subclusters enriched in 2 of 3 cohorts (Fig. 4B). We analyzed the CNV of each patient using inferCNV with the macrophages as reference cells and hepatocytes as observation cells (Supplementary Figs. S6–S8); we noted generally the CNV clones with more HBV expression indeed mostly consisted of subclusters 0 and 3, while the clones with less HBV expression consisted of mainly subcluster 2. We picked 3 representative patients (from 2 cohorts) with well-defined CNV clones and a sufficient (>50) number of HBV-positive cells, P114_SRP318499, P725_SRP318499, and P7_SRP278381, and generated an overall CNV for this set (Fig. 4C). The result shows that for patients in different cohorts, the clones with discernable, less ambiguous CNV patterns have a clear majority of cells with HBV expression compared to the other clones (Fig. 4C, green boxes).

We studied the CCI pattern of HBV–hepatocyte interactions and further grouped hepatocytes into HBV-positive (+) and normal (not HBV-positive) hepatocytes. CCIs in HBV-positive subclusters 0 and 3 had higher relative strength than normal ones, shown in Fig. 4D. Proteinase-activated receptor (PAR) signaling pathway is tested to be the most significant signaling pathway (Fig. 4D and E) across the HBV-enriched hepatocytes. PARs as the thrombin receptors are involved in thrombin-induced cell migration across a collagen transmembrane barrier [41]. Midkine (MK) is a growth factor that is tested to be a crucial role in HCC. It is involved in inflammatory responses, acts as an antiapoptotic factor, and blocks anoikic to promote metastasis [42].

### Identification of *H. pylori* reads in gastric cancer

**Figure 5:**
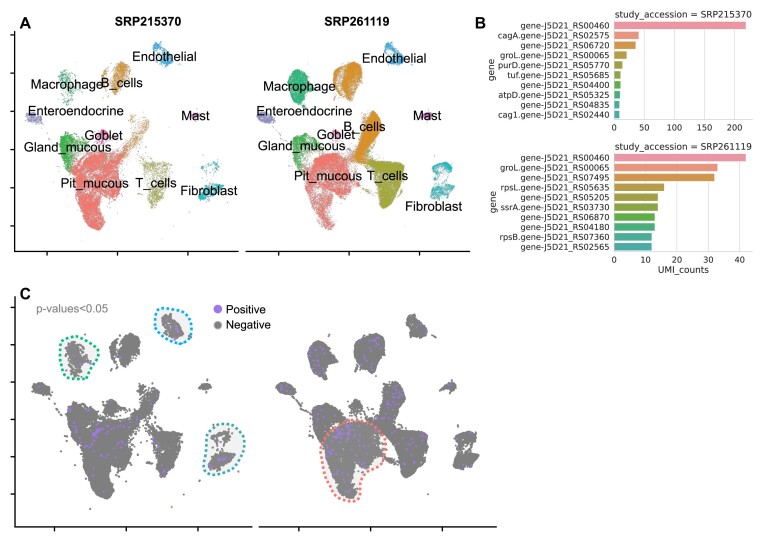
Viral calling meta-analysis results on the GC sample. (A) UMAP plot of the GC cells colored by cell-type annotations. (B) The transcript UMIs of *H. pylori* identified in SRP215370 and SRP2161119, separately. (C) UMAP plots of the intracellular *H. pylori. H. pylori–*positive cells are colored purple while other cells are gray.

We also conducted a meta-analysis of the 2 gastric cancer (GC) cohorts that have publicly available droplet scRNA-seq sequencing data, SRP215370 (Zhang et al. [11]) and SRP261119 (Kim et al. [12]). The former includes early GC samples (in which we only used the 2 confirmed *H. pylori*–positive patients), and the latter contains GC patient samples, the majority of which were known to be *H. pylori* positive. In total, we applied Vulture on the early GC or GC samples of 15 patients in the 2 cohorts, amounting to 125,845 after all QC filtering. We used the same approach as the above HCC case study for clustering and integration and labeled the cell types in line with Kim et al. [12] (SRP261119) (Fig. 5A). Microbial enrichment detection by cohort showed *H. pylori* enrichment in endothelial cells, fibroblasts, and macrophages in SRP215370 and enrichment only in pit mucous cells in SRP261119 (Fig. 5C and Supplementary Table S6). The difference is also present in the *H. pylori* virulence of 2 cohorts shown in Fig. 3B. CagA virulence gene is only detected in the Zhang et al. [12] cohort where cagA-positive strains are the strongest risk factor of gastric cancer [43].

## Discussion

In recent years, the rapidly growing single-cell study sources have become a gold mine for the reinvestigation of host–microbial interactions. But neither traditional practices on scRNA-seq studies nor the bioinformatics methods developed to detect microbes are capable of scalable meta-analysis for the public open data on the cloud. Alternatively, cloud computing has become an essential piece of equipment. Here, we present Vulture, a cloud-based scalable framework for calling microbial RNA on public scRNA-seq resources. Vulture was benchmarked on data originating from various tissues, generated with different scRNA-seq platforms, and deposited in different formats. It is tested to be highly scalable and cost-effective because it runs 200 analyses within a similar duration and is low cost compared to a single task. Moreover, running a single Vulture analysis on the local environment is substantially faster than previous methods. The reason is that Vulture provides an easily customizable combined reference. It performs read mapping once, while others map read to the host and then align unmapped reads to the microbe references, among other intermediate steps. Vulture runs faster by getting rid of complex preprocessing on unmapped reads. The combined reference is 30% larger than the host genome, but indexing from alignment tools can compensate for the increased reference size. Also, Vulture is user-friendly because it supports multiple platforms and multiple input formats. We demonstrated that Vulture can readily provide an effective solution for viral calling meta-analysis on large-scale public data.

We applied Vulture and scRNA-seq analysis to public COVID-19 BALF cells, HCC samples, and GC samples. The COVID-19 analysis revealed that Vulture can identify coinfections of unexpected pathogens. The HCC samples discovered a potential crucial relationship between CNV and intracellular HBV. All those cases indicate that Vulture is highly valuable to study unknown mechanisms and treatments by mining large-scale single-cell data.

However, there are several limitations of Vulture. A key hinder to Vulture meta-analysis is the permission of data. Serval atlas-level databases have strict access permission, which makes it hard to run cloud-based analysis on a large scale. Also, large-scale meta-data cleansing for raw sequencing files, which is essential to run parallel meta-analysis appropriately, is difficult because sequencing files are generated by different protocols. A prospective solution is to incorporate biomedical natural language processing (bioNLP) models and search engine technologies to subtract metadata for Vulture. Ultimately, Vulture automatically digs the gold mine of host–microbial interactions in big data.

In summary, Vulture is a cloud-based scalable framework for calling microbial RNA on public scRNA-seq resources. It is highly scalable, is cost-effective on the cloud, and substantially outperforms previous methods in local environments. We anticipate that Vulture will play a crucial role in the attempt to understand the unrevealed genetics of pathogenic diseases as the community gradually contributes to the increasing scale of single-cell data for host–microbial interactions.

## Availability of Source Code and Requirements

Project name: Vulture

Project homepage: https://github.com/holab-hku/Vulture

Project tutorial page: https://hiyin.github.io/vulture-user-tutorial

Operating system(s): For local version: Linux; for docker version: platform independent

Programming language: Python, Nextflow, and Perl

Other requirements: For local version: R = 4.0.5; DropletUtils >= v1.10.2; Docker = 20.10.21; STAR >= v2.7.9a (default) or cellranger >= 6.0.0 or Kallisto|bustools >= 0.25.1 or salmon|alevin >= v1.4.0. For cloud version: AWS batch.

License: MIT license

RRID:  RRID:SCR_024720

## Abbreviations

AWS: Amazon Web Services; BALF: bronchoalveolar lavage fluid; BAM: Binary Sequence Alignment Map; CCI: cell–cell interaction; CNV: copy number variation; GC: gastric cancer; HBV: hepatitis B virus; HCC: hepatocellular carcinoma; hMPV: human metapneumovirus; HSV: herpes simplex virus; scRNA-seq: single-cell RNA sequencing; UMI: unique molecular identifier.

## Supplementary Material

giad117_GIGA-D-23-00124_Original_Submission

giad117_GIGA-D-23-00124_Revision_1

giad117_Response_to_Reviewer_Comments_Original_Submission

giad117_Reviewer_1_Report_Original_Submission Yongxin Liu -- 6/20/2023 Reviewed

giad117_Reviewer_1_Report_Revision_1 Yongxin Liu -- 8/24/2023 Reviewed

giad117_Reviewer_2_Report_Original_Submission Jingzhe Jiang -- 7/8/2023 Reviewed

giad117_Reviewer_3_Report_Original_Submission Liuyang Zhao -- 7/18/2023 Reviewed

giad117_Reviewer_3_Report_Revision_1 Liuyang Zhao -- 8/27/2023 Reviewed

giad117_Supplemental_Files

## Data Availability

The datasets supporting the conclusions of this article are available in the SRA Run Selector repository [44]. They can be searched under the following project accessions numbers: SRP250732 [33], SRP279746 [14], SRP136347 [8], SRP278381 [9], SRP318499 [10], SRP215370 [11], and SRP261119 [12]. All supporting data and materials are available in the *GigaScience* GigaDB, database [45].

## Additional Files

**Supplementary Fig. S1**. Infrastructure of the Vulture application. (a) Describes the cloud services application and user workflow of Vulture on the cloud. (b) Describes the procedure of each Vulture application locally.

**Supplementary Fig. S2**. Consistency metrics among Vulture (or unfiltered) and other tested tools. (a, b) Venn diagrams showing the interaction of SARS-CoV-2–positive cells among all tested tools. Vulture (unfiltered) is the result of Vulture before EmptyDrops filtering. (c) Mean absolute error (MAE) of UMIs across the intersection of SARS-CoV-2–positive cells. (d) Pearson correlation of UMIs across the intersection of SARS-CoV-2–positive cells.

**Supplementary Fig. S3**. Viral calling meta-analysis results on the complete BALF sample. (a) A set of UMAP plots of the Bost et al. sample. The one on the left is a UMAP colored by subclustering annotations. The one in the middle is a plot of the SARS-CoV-2 infection. Infected cells are colored orange while other cells are gray. The one on the right is a plot of the HSV infection. Infected cells are colored blue while other cells are gray. (b) The marker genes expression across cell types to identify different cell types of the Liao et al. cohort. (c) The marker genes expression across cell types to identify different cell types of the Bost et al. cohort.

**Supplementary Fig. S4**. Ligand–receptor pairs with the strongest contribution among SARS-CoV-2–positive and hMPV-positive macrophages and monocytes in the COVID-19 BALF samples.

**Supplementary Fig. S5**. Viral calling meta-analysis results on the complete HCC sample. (a) A UMAP plot of the HCC sample; cells are colored by subclustering annotations. (b) UMAP plots of the HBV infection. Infected cells are colored blue while other cells are gray. (c) The marker gene expression across cell types to identify different cell types. (d) UMIs of the detected transcripts from HBV.

**Supplementary Fig. S6**. inferCNV diagram of SRP278381.

**Supplementary Fig. S7**. inferCNV diagram of SRP318499.

**Supplementary Fig. S8**. inferCNV diagram of SRP136347.

Supplementary Tables 1-6.xlsx. Cell type specific enrichment scores of microbes across cohorts.

Supplementary Table 7.csv.
