# Supplementary material for: Vulture: cloud-enabled scalable mining of microbial reads in public scRNA-seq data
Source: Gigascience. 2024 Jan 9;13:giad117. doi: 10.1093/gigascience/giad117 (PMC10776309; doi:10.1093/gigascience/giad117)
Supplement: giad117_GIGA-D-23-00124_Revision_1 [file giad117_giga-d-23-00124_revision_1.pdf]

GigaScience

Vulture: Cloud-enabled scalable mining of microbial reads in public scRNA-seq data

--Manuscript Draft--

Manuscript Number:	GIGA-D-23-00124R1
Full Title:	Vulture: Cloud-enabled scalable mining of microbial reads in public scRNA-seq data
Article Type:	Research
Funding Information:	Innovation and Technology Commission - Hong Kong / Dr Joshua W.K. Ho
Abstract:	The rapidly growing collection of public single-cell sequencing data has become a valuable resource for molecular, cellular, and microbial discovery. Previous studies mostly overlooked detecting pathogens in human single-cell sequencing data. Moreover, existing bioinformatics tools lack the scalability to deal with big public data. We introduce Vulture, a scalable cloud-based pipeline that performs microbial calling for single-cell RNA sequencing (scRNA-seq) data, enabling meta-analysis of host-microbial studies from the public domain. In our benchmarking experiments, Vulture is 88-66% faster than local tools (PathogenTrack and Venus), and 41% faster than the state-of-the-art cloud-based tool Cumulus, while achieving comparable microbial read identification. In terms of the cost on cloud computing systems, Vulture also shows a cost reduction of 83% ($12 vs $70). We applied Vulture to two COVID-19, three hepatocellular carcinomas (HCC), and two gastric cancer human patient cohorts with public sequencing reads data from scRNA-seq experiments and discovered cell-type specific enrichment of SARS-CoV2, hepatitis B virus (HBV), and Helicobacter pylori (H. pylori) positive cells, respectively. In the HCC analysis, all cohorts showed hepatocyte-only enrichment of HBV, with cell subtype-associated HBV enrichment based on inferred copy number variations. In summary, Vulture presents a scalable and economical framework to mine unknown host-microbial interactions from large-scale public scRNA-seq data. Vulture is available via an open-source license at https://github.com/holab-hku/Vulture.
Corresponding Author:	Joshua W. K. Ho, PhD<br>University of Hong Kong<br>Hong Kong, HONG KONG
Corresponding Author Secondary Information:	
Corresponding Author's Institution:	University of Hong Kong
Corresponding Author's Secondary Institution:	
First Author:	Junyi Chen
First Author Secondary Information:	
Order of Authors:	Junyi Chen
	Danqing Yin
	Harris Y.H. Wong
	Xin Duan
	Ken H.O. Yu
	Joshua W.K. Ho
Order of Authors Secondary Information:	
Response to Reviewers:	Response letter to reviewer comments<br><br>Reviewer #1:<br>Comment 1: “The software, tested data and results are required to be uploaded on GitHub for peers to use, and conda and/or docker installation modes are recommended for software with complex dependencies. We will take software Star,

Powered by Editorial Manager® and ProduXion Manager® from Aries Systems Corporation

Fork, and downloads of GitHub as one of the audience indicators … Now this project is only 2 stars. You need more people to take part in and interest in this project."
Response 1: We have now released the Vulture Docker image on Docker hub (https://hub.docker.com/repository/docker/junyichen6/vulture/general) with a corresponding section "Running Vulture with docker image" (https://hiyin.github.io/vulture-user-tutorial/1.%20Running%20on%20local%20computer/4_Docker/) in the Vulture tutorial. We are confident that all the changes in the revised version will improve the overall popularity of Vulture, especially after it is published.

Comment 2: "…. I found the GitHub links: https://github.com/holab-hku/Vulture. However, the readme.md show pipeline on AWS cloud. If I not have an AWS, how can I run it in my server…."
"Software installation and User tutorial are required in Readme.md or Wiki in GitHub. Please provide step by step protocol to deploy it in the laptop or server."
"A video of software download, installation, operation, and result display is required with a computer or server without any related software installed, to make sure that any new user can perform the whole process according to the tutorial."
Response 2: To further enhance the usability of Vulture applications beyond AWS, we have substantially updated the documentation to include step-by-step instructions to run Vulture on local servers (https://hiyin.github.io/vulture-user-tutorial/1.%20Running%20on%20local%20computer/1_Quickstart/). We have created a comprehensive video tutorial in the page covering software download, installation, operation, and result display. Starting from scratch, users should be able to perform the entire process successfully. The combination of command line instruction, video tutorial, and docker instruction mentioned in Response 1, should enhance the usability of Vulture beyond AWS.

Comment 3: "The software is required to be posted on twitter and other social media, you can contact @ iMetaScience, @microbe_article etc. to get help in tweet or retweet. The number of Retweet, Like and View as one of the audience indicators."
Response 3: We will of course actively promote Vulture on social media and other channels after the paper has completed the peer review process.

Comment 4: "Chinese is largest single langue science society. Provide the Chinese tutorial and video presentation of the software, contact meta-genome Official account for help to promote. The Number of readers, share and favorite also one of the audience indicators."
Response 4: Adding Chinese language support is our plan for the next stage of development. In our experience, all Chinese users have no problem reading and using our software packages.

Comment 5: "According to the feedback from users in all over the world, the author continuously maintains and optimizes the method to ensure its availability, ease of use and advancement."
Response 5: As seen by the effort made during this round of revision, of course we will make continuous efforts in maintaining the Vulture repository to ensure its availability, user-friendliness, and advancement.

Comment 6: "The software name should be unique, which is convenient to count the real users through all available resources (such as QIIME, ImageGP, and EasyAmplicon). However, the name vulture is unacceptable, due to million of hits in Google scholar."
Response 6: There are indeed many widely-used bioinformatics tools with common names such as bowtie and cufflinks. These tools gained recognition within the bioinformatics community due to their performance, reliability, and wide adoption. 'Vulture' is a unique name for a bioinformatics tool and will be easily recognizable when it is widely used.

Comment 7: "The figures in your papers are diversity. However, I cannot find enough visualization function in your pipeline. The pipeline for integrated software is easy, the specific and diversity visualization plan is difficult. All the authors want their analysis result is ready-to-published."
Response 7: The source code to support the generation of individual figures in this

	paper will be available on the GigaDB after being published. The functionalities provided by Vulture are already on par with other tools such as Viral-Track, PathogenTrack and Venus. I am sure most bioinformaticians can handle basic visualisation using the example source code we provide.<br><br>Comment 8: "Why only focus on the virus? Can this pipeline to generate all the microbiome, which is more interest and overview of the microbes."<br>Response 8: Indeed Vulture can be used to identify any species in which a reference genome sequence can be provided. In the current version, Vulture's database includes bacteria, archaea and viruses. To clarify this point, we have changed the title of this manuscript to 'Vulture: Cloud-enabled scalable mining of microbial reads in public scRNA-seq data'.<br><br>Comment 9: "The figures need to be improved. Such as ImageGP (10.1002/imt2.5) can generate high quality figures and with reproducible scripts."<br>Response 9: We have uploaded high-resolution figures in vectorized format. Additionally, we uploaded the figure reproduction scripts to the GIGA Science FTP server, which will be made available on GigaDB after the paper is published.<br><br>Comment 10:" Too many tables. Please move then as the supplementary table."<br>Response 10: Tables. 2 to 7 are supplementary information to complement results presented in Fig. 3c, 4b, and 5c. We have made the necessary adjustments and moved these tables to Supplementary Table S1 to S6.<br><br>Comment 11: "The structure of the results appears not in concise. The authors should have presented their findings in around 3 mainly sections will be better. Some unimportant result in supplementary figures. In order to facilitate readers to better grasp the key points."<br>Response 11: We have edited the result session, particularly the subsection "Vulture enables cloud-based discovery of Metapneumovirus reads in COVID-19 BALF samples". The revised section describing Fig. 3 is more concise.<br><br>Reviewer #2:<br>Comment 12: In this study, Chen et al. introduce Vulture, a scalable cloud-based pipeline that performs microbial calling for single-cell RNA sequencing (scRNA-seq) data, enabling meta-analysis of host-microbial studies from the public domain. And they further applied Vulture to COVID-19, HCC, and gastric cancer human patient cohorts with public sequencing reads dataand discovered cell-type specific enrichment of SARS-CoV2, hepatitis B virus (HBV), and H. pylori positive cells. Generally speaking, this study is innovative, has good application potential, and can better assist the work of single cell research from the point of view of infection. I only a few minor questions that need the author to reply:<br><br>1. "Background: The first appearance of H. pylori should be replaced with its full name."<br>Response 12: Fixed.<br><br>Comment 13. "Methods-Downstream analysis of scRNA-seq samples: Why use different tools (SCANPY/Seurat, BBKNN/Harmony) to analyze different datasets instead of using the same tool to analyze different datasets?"<br>Response 13: We initially employed different tools, such as SCANPY and Seurat, for analyzing distinct datasets due to scalability considerations. Given that the cohort of COVID-19 samples contains a larger number of cells compared to the HCC cohort, a Python-based pipeline was chosen for efficient analysis. In response to your question and to present more consistent results, we have revised our analysis. We now employ the Seurat/harmony pipeline for the downstream analysis and updated Fig. 3c, as well as Supplementary Table S1, to enhance the coherence of findings across datasets.<br><br>Comment 14. "Cell-type enrichment of microbial UMI: format error of formula."<br>Response 14: Fixed.<br><br>Comment 15. "Analyses-Page 11: "The statistical test identified that SARS-CoV-2 is

enriched (p-value < 0.05) in epithelial cells, neutrophils, and plasma B cells (Fig. 3d and Table. 2)". It is best to highlight p < 0.05 data points in other colors rather than red squares. Why are there no p < 0.05 square in fig. 3e?"
Response 15: We have revised the color scheme in Fig. 3c, 3d, 4b, and 5c. Currently, virus/microbe-enriched cell types are highlighted by circles. The annotation for hMPV enrichment in Fig. 3d is added.

Comment 16. "Fig. 2a and 2b: There are 8 colors in figure 2a, however only 4 figure legend were showed. What do the four light-colored bar mean? And the same to Fig 2b."
Response 16: We have revised Fig. 2 by merging Fig. 2 a, b to a single Fig. 2a to ensure that the comparison is accurately explained. The pastel-colored and saturated-colored bars in Fig. 2a represent the total time and the individual time of a computing process, separately. A legend to describe the color bar has been added to the figure. The total and individual time for the mapping process in Cumulus is demonstrated as line plots. The star-marked and round-marked dash lines in Fig. 2a represent the total time and the averaged individual time of the Cumulus marking time, separately.

Reviewer #3:
The authors aim to develop Cloud-enabled approaches for detecting viral reads in public single-cell RNA sequencing (scRNA-seq) data. This study makes a significant contribution to the identification of viruses and bacteria in public scRNA-seq data. Although the outcomes are satisfactory, the novelty of the proposed methods is limited. To date, no evidence has been provided to demonstrate their superiority over recently published methods (such as PathogenTrack and Venus, et al) when executed on a local machine. There are also several issues that need to be further addressed, as highlighted below:

Comment 17." The documentation available on the GitHub pipeline does not explain how to utilize the latest virus database or how to incorporate a user's custom database. Because the virus database is updated very quickly now. It might be more appropriate if the author updates the database promptly or if one can customize and create their own database."

Response 17: In fact, Vulture includes a dedicated function for constructing genomes. It downloads the latest list of microbes from both the ViruSITE and NCBI prokaryotes databases. These databases are frequently updated to ensure the inclusion of the most recent data. Our function is designed for users to maintain an up-to-date reference genome. We have also provided an enhanced step-by-step tutorial (https://hiyin.github.io/vulture-user-tutorial/3.%20Building%20your%20reference%20genome/1_Build/) to provide a more comprehensive demonstration of the genome construction process. Also, we will regularly upload the latest version of the reference genome to our repository to provide access to the most recent data for users.

Comment 18. "Figure 2a only has an overall comparison graph, it can be improved by adding detailed comparison graphs with Cumulus, PathogenTrack and Venus."
"Figure 2b. The persuasiveness is not enough, it would be better to compare several pipeline platforms with similar functionalities or compare some specific steps, such as the four steps in figure 2a. By the way, all of these comparisons use comparison software developed by other same researchers, so please provide a detailed description of why the author's method is faster?"
Response 18: We have revised Fig. 2 by merging Fig. 2 a, b to a single figure mentioned in Response 15. The line plot in Fig. 2c has become a bar plot in Fig. 2b. It includes detailed individual steps duration of comparison among viral calling methods on one single analysis with different input file sizes. We have visualized 3 steps (Map, Analysis, and Filter) from Vulture, 2 steps (Map by Cellranger and Count by Pathogentrack) from Pathogentrack and Venues as a whole. The total duration of tasks is presented in stacked bar plots.
We have discussed the reason that makes Vulture run faster on local machines in the Discussion session. Vulture performs only one read mapping to the host-microbial

Powered by Editorial Manager® and ProduXion Manager® from Aries Systems Corporation

	reference genome, while others map reads to the host genome and then process a second round of read mapping to the microbe or virus references. For the cloud version, we discussed in the Result session that Vulture takes advantage of the AWS Batch spot capacity optimization technique. It ensured a higher availability of computational resource allocation, a larger number of concurrent tasks, and less response time for pending tasks compared to the unconfigurable Terra platform adopted by Cumulus.<br><br>Comment 19. “Figure 3c can be created with microbial clustering and non-microbial clustering to highlight the impact of virus identification on classification results.”<br>Response 19: We have revised the color scheme in Fig. 3c, 3d as described in the response to reviewer 2’s comment 2. Fig. 3 c, d and e are now spitted by sars-cov-2 positive and hMPV positive to highlight the impact of virus identification on classification results.<br><br>Comment 20. Fig. S1 It should be the "Quality control on read level".<br>Response 20: Fixed.
**Additional Information:**	
**Question**	**Response**
Are you submitting this manuscript to a special series or article collection?	No
**Experimental design and statistics**<br><br>Full details of the experimental design and statistical methods used should be given in the Methods section, as detailed in our Minimum Standards Reporting Checklist. Information essential to interpreting the data presented should be made available in the figure legends.<br><br>Have you included all the information requested in your manuscript?	Yes
**Resources**<br><br>A description of all resources used, including antibodies, cell lines, animals and software tools, with enough information to allow them to be uniquely identified, should be included in the Methods section. Authors are strongly encouraged to cite Research Resource Identifiers (RRIDs) for antibodies, model organisms and tools, where possible.<br><br>Have you included the information requested as detailed in our Minimum	Yes

Standards Reporting Checklist?	
**Availability of data and materials**<br><br>All datasets and code on which the conclusions of the paper rely must be either included in your submission or deposited in publicly available repositories (where available and ethically appropriate), referencing such data using a unique identifier in the references and in the "Availability of Data and Materials" section of your manuscript.<br><br>Have you have met the above requirement as detailed in our Minimum Standards Reporting Checklist?	Yes

Manuscript Click here to access/download;Manuscript;Main_2023_11_17_revised

Vulture: Cloud-enabled scalable mining of microbial reads in public scRNA-seq data

Junyi Chen[1,2], Danqing Yin[1,2], Harris Y.H. Wong[1], Xin Duan[1], Ken H.O. Yu[1,2]^, and Joshua W. K. Ho[1,2]^

[1]Laboratory of Data Discovery for Health Limited (D[2]4H), Science Park, Hong Kong SAR, China

[2]School of Biomedical Sciences, Li Ka Shing Faculty of Medicine, The University of Hong Kong, Pokfulam, Hong Kong SAR, China

^Co-corresponding authors: Ken H. O. Yu (yuken@hku.hk), Joshua W. K. Ho (jwkho@hku.hk)

ORCID iDs:

Junyi Chen [0000-0002-9992-7273]; Danqing Yin [0000-0003-0448-4754]; Harris Y H Wong [0000-0003-4464-0547]; Xin Duan [0000-0001-5410-3847]; Ken H O Yu; Joshua W K Ho [0000-0003-2331-7011];

Abstract

The rapidly growing collection of public single-cell sequencing data has become a valuable resource for molecular, cellular, and microbial discovery. Previous studies mostly overlooked detecting pathogens in human single-cell sequencing data. Moreover, existing bioinformatics tools lack the scalability to deal with big public data. We introduce Vulture, a scalable cloud-based pipeline that performs microbial calling for single-cell RNA sequencing (scRNA-seq) data, enabling meta-analysis of host-microbial studies from the public domain. In our benchmarking experiments, Vulture is 88-66% faster than local tools (PathogenTrack and Venus), and 41% faster than the state-of-the-art cloud-based tool Cumulus, while achieving comparable microbial read identification. In terms of the cost on cloud computing systems, Vulture also shows a cost reduction of 83% ($12 vs $70). We applied Vulture to two COVID-19, three hepatocellular carcinomas (HCC), and two gastric cancer human patient cohorts with public sequencing reads data from scRNA-seq experiments and discovered cell-type specific enrichment of SARS-CoV2, hepatitis B virus (HBV), and *Helicobacter pylori* (*H. pylori) positive* cells, respectively. In the HCC analysis, all cohorts showed hepatocyte-only enrichment of HBV, with cell subtype-associated HBV enrichment based on inferred copy number variations. In summary, Vulture

presents a scalable and economical framework to mine unknown host-microbial interactions from large-scale public scRNA-seq data. Vulture is available via an open-source license at https://github.com/holab-hku/Vulture.

Keywords

Cloud computing; single cell; COVID-19; HCC; Virus;

Background

Pathogenic diseases are considered a significant threat to global health, such as severe acute respiratory syndrome coronavirus 2 (SARS-CoV-2) in coronavirus disease 2019 (COVID-19), hepatitis B virus (HBV) and hepatitis C virus (HCV) in hepatocellular carcinoma (HCC) [1], and *H. pylori* in gastric cancer (GC) [2]. Single-cell or single-nucleus RNA sequencing (sc/snRNA-seq) has reformed the investigation of complex diseases and contributed to discoveries of host-microbial interaction mechanisms [3]–[7]. Due to the rapidly maturing scRNA-seq technologies, the exponentially growing public scRNA-seq data resources have become a gold mine for conducting *in silico* investigations toward host-microbial interactions.

In the current practice of scRNA-seq data processing, a key concern is the selection of the reference genomes when quantifying the reads. Most studies only align reads to the host genome or focus on limited microbial genomes [8]–[12]. This practice systematically risks missing either the known or unknown host-microbial interactions in the datasets. It is therefore worthwhile to perform re-analyses of existing public scRNA-seq data on the cloud to uncover the breadth of these interactions. According to the Human Cell Atlas [13] Data Portal, as of Dec. 2022 there are an estimated 12.3M cells from 2,400 specimens, totalling 38.1 TB in file size of published human cellular droplet-based scRNA-seq data. As more and more files become available, cloud computing becomes increasingly enticing as the choice for performing large-scale re-analyses that can leverage huge amounts of computational

resources without the need to purchase or maintain expensive hardware and avoid the transfer of large amounts of data.

Several tools have been developed for the identification of microbial reads in human scRNA-seq data on local machines. Viral-Track [14] is an existing computational pipeline that detects viral-host interactions in droplet scRNA-seq data by scanning host-unmapped reads for the presence of viral RNA. Based on a similar schema, Zhang et al. and Lee et al. developed PathogenTrack [15] and Venus [16] which have added capabilities. PathogenTrack can quantify bacteria in addition to viruses; the tool was benchmarked to be mostly correlated in microbial unique molecular identifiers (UMIs) called as and faster in run time than Viral-Track [15]. Venus identifies viruses only but has another module to discover viral integration sites. However, due to the number of steps and certain tool choices in these pipelines, their scalability can still be improved.

These off-the-shelf microbial calling methods for scRNA-seq are developed as command-line tools running on local computing environments, which may eventually struggle with the scale of published data on the cloud. Only with cloud computing can we obtain the scRNA-seq big data as well as leverage a huge amount of computational resources without the maintenance of expensive devices. Previously, we [17] developed the cloud-based Falco framework for scalable scRNA-seq analysis. On two public scRNA-seq datasets, it was 2.6-145.4 times faster than running on the local computing environments. Li et al. [18] developed a scalable scRNA-seq analysis framework based on the Terra platform called Cumulus afterwards. With Cumulus, Delorey et al. [19] performed COVID-19 scRNA-seq dataset analysis on 420 specimens from 11 organs in 2021. In 2022, Edgar et al. [20] developed the Serratus framework. They reviewed 5.7 million transcriptome sequencing (RNA-seq) data for RNA-dependent RNA polymerases and identified more than 105 novel RNA viruses. However, Falco only supported the Smart-Seq protocol since 2017 which is insufficient nowadays. Also, neither the Cumulus nor the Serratus pipeline focus on the host-microbial scRNA-seq analysis.

To perform a large-scale meta-analysis of public scRNA-seq data, we developed Vulture, which to our knowledge is the first cloud-based scalable framework for discovering microbial reads in public scRNA-seq data. It can be executed either on the cloud container services in parallel or in a local

environment. Our tool provides an easily modifiable, host-microbial combined reference that standardizes the gene transcript annotations of human and known human-host viruses and bacteria. Additional features of our Vulture are the support of multiple formats of raw sequencing file inputs, and the quality control metrics of the identified intracellular microbial UMIs. We benchmarked the scalability and cost-effectiveness of our tool, and show it outperforms existing solutions. With Vulture, we re-analyzed cohorts of COVID-19, hepatocellular carcinoma, and gastric cancer with public raw sequencing data of droplet scRNA-seq and examined the host-microbial interactions of SARS-CoV-2, HBV, and *H. pylori*, respectively. Specifically, we detected an upregulation of chemokine receptor crosstalk along with the co-infection of SARS-CoV2 and human metapneumovirus (hMPV) from a COVID-19 bronchoalveolar lavage fluid (BALF) sample and a potential HBV-induced copy number variations from an HCC sample. The result shows the utility of viral calling to the full set of known host microbes.

Methods

Cloud infrastructure of Vulture on AWS batch infrastructure with Nextflow

The cloud framework of Vulture is described in **Supplementary Fig. S1a**. Vulture applications on the cloud are constructed in Docker containers. A container is a lightweight software unit that packages all our procedures and dependencies for sequence alignment, quality control, and downstream analysis. Containerized Vulture applications are managed by the Amazon Web Services (AWS) Batch service. AWS Batch is a batch management capability to efficiently run a huge amount of batch computing jobs on AWS. The Batch is a job scheduler composed of four elements including Compute Environments, Job queues, Job definitions, and Jobs. The Compute Environment specifies the computational resources required for a type of task. We applied the SPOT_CAPACITY_OPTIMIZED allocation strategy in Batch to prioritize the use of spot instances in Compute Environment. The Job Queue maps the Vulture pipeline task to one or more Compute Environments. The Job Definition is a template that assigns the Docker image to be employed in running a particular task along with its

parameters such as the number of CPUs, the amount of memory, and other configurations. The Jobs binds a Job Definition to a specific Job Queue and executes the task command in the Docker container. In the Vulture pipeline, Job definitions and execution of Jobs are controlled by Nextflow, a language that streamlines the deployment of workflows on the commercial cloud and clusters. Nextflow creates the required Job Definitions and Jobs as needed. Each Job can use a different queue and Docker image. The Vulture container is published in DockerHub and Elastic Container Registry (ECR) that are accessible from the instances run by Batch. The Simple Storage Service (S3) bucket is where the input, output, and working directory of the Vulture pipeline are stored during execution.

Construction of host-microbe combined reference genome

The first step of Vulture is to construct reference genomes and corresponding annotations for the host (human, in this study) and host-infection viruses and bacteria. We use 245 distinct human-host prokaryotes curated by the NCBI Genome [21] and 529 human-host viral species from viruSITE [22] which together with the human reference genome hg38 form a combined reference set. The set is a collation of the reference genome fasta sequences and exon/transcript/gene gtf annotations of all species used. Non-host exons with a minimap2 (RRID:SCR_018550) [23] alignment to the host genome were removed due to ambiguity. The combined host-microbe reference genome was indexed using the ***genomeGenerate*** module from the STARsolo (RRID:SCR_021542) [24] tool.

Quantifying reads from scRNA-seq data to count matrices

Vulture supports a variety of alignment algorithms to quantify scRNA-seq sequences with the constructed combined reference genome. Users can select STARsolo [24] (default), Cell Ranger [25], Kallisto | bustools [26], and Alevin [27]. Sequence data can be quantified as a two-dimensional Unique Molecular Identifiers (UMI) count matrix of cells × genes and the corresponding Binary Sequence Alignment Map (BAM). BAM files are only generated if STARsolo or Cell Ranger is selected.

Quality control of the mapped microbial reads and count matrix

After obtaining the results of the sequence alignment, we also perform additional quality control steps to increase the likelihood that the viral sequences found are intracellular and reliable. Vulture utilizes the EmptyDrops [28] algorithm to filter out the droplets with non-cellular ambient RNA. We then optionally perform various quality analyses on the BAM files documenting the sequence alignments, including multi-mapping of the resulting sequences to host or non-host genes and reads dispersion, which is the extent of unique positions the reads are aligned on a given transcript.

Downstream analysis of scRNA-seq samples

The meta-analysis of the Vulture processed results is composed of several procedures. We applied Seurat (RRID:SCR_007322) [30] for the COVID-19, HCC and GC samples to perform scRNA-seq processing, and clustering, respectively. For the batch effects removal across different cohorts, we applied Harmony [32] for the COVID-19, HCC and GC samples. CellChat (RRID:SCR_021946) [33] is applied to calculate the ligand-receptor interactions among the annotated cell types. Copy number variation (CNV) inference and clone identification for the HCC sample is analyzed by the inferCNV (RRID:SCR_021140) [34] package.

Cell-type enrichment of microbial UMI

We follow the idea in [19] to calculate the cell-type-specific enrichment score of intracellular microbes. The reason is that the number of microbial UMIs is small and their differences across cell types are difficult to observe. The enrichment score for cluster C in the clustering of cells is computed as follows:

$$Enrichment(C) = log(\frac{N_V^C + \varepsilon}{N_V \times P_C + \varepsilon})$$

N_V^C is the number of microbe-positive cells in cluster C, N_V is the number of microbe-positive cells in the whole cohort, P_C is the proportion of the total number of cells in cluster C out of the total number of cells in the cohort, and ε is a small float to avoid zero subtractions.

The p-value of cell-type-specific enrichments of intracellular microbes was calculated by randomly permuting the identical number of microbe's positive annotations to all cell types 10,000 times. The

empirical p-value is the proportion of permutations that get an enrichment score not less than the actual score in the cohort out of 10,000 times. We also perform the FDR correction on the empirical p-value.

Analyses

Vulture: a cloud-based microbial calling framework for public scRNA-seq data

The architecture of Vulture is shown in **Fig. 1**. Vulture is composed of a bioinformatics analysis container, a cloud platform, and a workflow management tool. The container defines five main processes for performing microbe calling for sc/snRNA-seq data: sequence data retrieval, human-microbe combined reference construction (optional), reads alignment, quality control, and downstream analysis (optional). Detailed implementations are listed in the Method section and **Supplementary Fig. S1a**. Vulture receives two major inputs by default: 1) the sequencing files, and 2) microbe genome files. The input sequencing files can be a set of run accession numbers (prefixed by SRR) from SRA, a set of Amazon S3, or HTTP download URLs. Both fastq and bam files are supported. As for the input of combined reference, we provide a default host-microbe reference covering human and all human-host microbe genomes. Users can also build their custom combined genome by inputting a list of microbe genome accession numbers based on viruSITE or NCBI.

Vulture utilizes cloud computing to provide a fast, scalable, and cost-effective, viral calling framework without the need for hardware maintenance. Vulture is built on the AWS Batch service natively, which efficiently runs a huge amount of computing jobs while optimizing compute resources. At the same time, Vulture is implemented by a docker container and can be easily run on local servers or other cloud platforms. We applied Nextflow (RRID:SCR_024135), a workflow management language to deploy complex parallel workflows of containers on clouds and clusters. The cloud architecture of Vulture is described in the Method section and **Supplementary Fig. S1b**. Through an AWS Batch and Nextflow, users can run thousands of viral calling tasks for public scRNA-seq data in parallel with simple configuration inputs.

Runtime performance and cost-effectiveness of Vulture on the cloud

To validate and benchmark the scalability of Vulture on the cloud, we tested it through the public COVID-19 scRNA-seq data by Bost et al. [14] consisting of up to 400 individual fastq files. Execution duration (pastel) and vCPU time (saturated) from retrieving files to bam analysis of running 25 to 200 parallel tasks are recorded in **Fig. 2a**. Vulture analysis on 200 fastq files within 48 minutes, reaching a speed up (compared to running 200 single tasks sequentially) of 155x, showing that it is highly scalable. We also compare Vulture to another cloud-based tool Cumulus [18] in read mapping on an identical pre-built host-microbe genome because Cumulus did not natively support viral calling tasks. **Fig. 2a** indicates that Vulture outperformed Cumulus nearly twofold in total duration (saturated). Costing $12, Vulture runs 200 alignment tasks in 20 minutes, while Cumulus needs $69 to run 200 samples in 32 minutes. Vulture takes advantage of the AWS Batch spot capacity optimization technique. Its utilization of spot instances reduced the cost of running viral calling pipelines. Also, it ensured the availability of computational resource allocation, maximizing the number of concurrent tasks, and minimizing the response time of pending tasks.

Performance of Vulture on the local environment against off-the-shelf tools

We also tested Vulture local command line tools against Venus and PathogenTrack [15], [16] using three COVID-19 scRNA-seq samples with different sizes: a 1GB small sample (SRR12570205) from Bost et al. [14], a 67 GB medium sample (SRR11537951) and a 141 GB large sample (SRR11181956) from Liao et al. [35]. **Fig. 2b** indicated that Vulture is the most computationally effective method among the three. On medium and large samples, Vulture took 83 and 187 minutes to finish the analysis. It was two to threefold faster than Venus (463 and 538 minutes respectively) and nine to tenfolds faster than PathogenTrack (832 and 2214 minutes respectively). Besides, we tested the consistency among methods on three datasets by measuring the intersection of SARS-CoV-2positive cells in **Fig. 2d**. The result of Vulture before filtering the empty droplets [28] named “Vulture (unfiltered)” is also added to the comparison. Since Vulture (unfiltered) is a superset of the Vulture result, the intersection between the two is the Vulture set in the figure. **Fig. 2d** indicates that Vulture reaches consensus with the others

in most cases because most major sets are intersections with other methods. After all, the intersection of the three methods (which is usually the smallest) is the third largest in the test. Vulture's result is most consistent with Venus with the largest set of intersections. Vulture (unfiltered) is the most sensitive with the largest number of SARS-CoV-2 positive cells (**Fig. 2d**) that cover most of the cells identified by others (**Supplementary Fig. S2a**). It is the quality control step that filtered out many empty droplets. On 135 cells, the intersection of the three, we calculated the mean absolute error (MAE) and Pearson correlation of SARS-CoV-2 viral UMI counts across methods in **Supplementary Fig. S2c and d**. The MAE between Vulture, Venus, and PathogenTrack are smaller than 1, showing that Vulture consistently generated microbial calling results compared to state-of-the-art methods in a faster manner.

Vulture enables cloud-based discovery of Metapneumovirus reads in COVID-19 BALF samples

SARS-CoV-2 infection has been identified to be the source of the worldwide COVID-19 pandemic since 2019. Many aspects of how the viral-host interaction have remained unrevealed. There is a major interest in identifying co-infection of other pathogens in COVID-19 patients. Therefore, we applied Vulture on the cloud on BALF samples from the Sequence Read Archive (SRA) to call viruses. We performed a meta-analysis on the Liao et al. [35] cohort from China (SRP250732) and the Bost et al. cohort [14] (SRP279746) from Israel. We ran a downstream analysis on BALF samples for two cohorts, totalling 51,338 and 991,722 cells after all QC filtering, respectively. After preprocessing and clustering the cell types were defined based on marker genes from Bost et al. and Liao et al. (**Supplementary Fig. S3b and c)**. Given the fact that SARS-CoV-2 UMIs in scRNA-seq data are relatively low and imbalanced, a statistical test (see methods) [19] is performed to estimate cell-type-specific enrichment of SAR-CoV-2 infection.

The combined microbe-host genome in Vulture includes a comprehensive set of human-host microbes to identify co-infections or unaware microbes. Vulture revealed human metapneumovirus (hMPV) co-infection with SARS-CoV-2 in Liao et al. [35] cohort (SRP250732), and unexpected Herpes simplex

viruses (HSV) in the Bost et al. cohort [14] (SRP279746), consistent with previous findings [14]. UMIs for different viral transcripts are in **Fig. 3a and b** Cell type visualization of BALF cells in Liao cohort is in **Fig. 3c,** SARS-CoV-2 and hMPV presence in **Fig. 3d and e.** UMAP plots for Bost cohort and SARS-CoV-2 and HSV presence in **Supplementary Fig. S3a.** Statistical tests found SARS-CoV-2 enriched (p-value < 0.05) in epithelial cells, neutrophils, and plasma B cells (**Fig. 3d and Supplementary Table. S1**), hMPV enriched in CD8+ T cells, NK cells, macrophages, and monocytes (**Fig. 3e and Supplementary Table. S3**). **Fig. 3e** shows a separate monocyte subtype with hMPV infections. Therefore, we compare the differential expressed genes between the hMPV-enriched monocytes/macrophages to the hMPV-negative monocytes/macrophages. Differentially expressed genes for the virally infected subtype are shown in **Supplementary Table S7.** S100A8/S100A9 up-regulated in hMPV-enriched macrophages/monocytes, involved in neutrophil-related inflammation [36]. FCN1 up-regulated, encoding a complement cascade member [3]. IDO1 up-regulated, murine coronavirus infection activates AhR independently, affecting cytokines [37]. g:Profiler [38] identified functional enrichment of the top 100 up-regulated genes in hMPV-enriched subtypes. **Fig. 3f** results indicate subtypes' patterns drive innate immune, cytokine responses. Interferon-gamma (IFN-γ) response activates IFN response in alveolar macrophages, recruits monocyte-derived alveolar macrophages and forms an inflammatory signaling circuit [36].

We analyzed SARS-CoV-2 and hMPV co-infection cell-cell interactions (CCI) using CellChat （RRID:SCR_021946） [33]. Cell types are divided into subgroups: SARS-CoV-2 positive (+-), hMPV positive (-+), double positive (++), and normal cells. Virus-infected macrophages and hMPV-infected monocytes showed stronger CCI than normal cells (**Fig. 3g)**. The Chemokine Signaling Pathways (CCL and CXCL) were significant (**Fig. 3h and i**) in virally infected macrophages and hMPV-infected monocytes. These pathways play a role in severe COVID-19, SARS, and MERS [39]. Key ligand-receptor pairs in the CCL pathway are CCL3/5/7/8 binding to CCR1/5 (**Supplementary Fig. S4)** driving monocyte recruitment, subsequent macrophage differentiation, and activating more immune cells, causing lung epithelial damage [40]. The macrophage migration inhibitory factor (MIF) pathway

predicts the outcome of acute respiratory distress syndrome (ARDS) and hallmarking severe COVID-19 [41]. The GALECTIN pathway also exhibited strong interactions, influencing infection consequences [42]. ANNEXIN and SPP1 (**Fig3. h**) also contribute strong CCI among monocytes and macrophages.

Cloud-based meta-analysis reveals an HBV-associated CNV signature in HCC

Another advantage of having a cloud-based framework is that it facilitates the integration of multiple data sets that are already in the same repository on the cloud. We performed a meta-analysis of the three public hepatocellular carcinoma (HCC) cohorts with droplet scRNA-seq sequencing data, SRP278381, SRP136347, and SRP318499. We ran Vulture on the HCC samples of 24 patients in these cohorts, totalling 421,780 cells following all QC filtering. After clustering and integration, the cell types were defined based on marker genes from Sharma et al. (SRP278381) (**Supplementary Fig. S5c**). Microbial enrichment detection (**Supplementary Table. S4**) on each of the cohort's indicated hepatocytes are the only cell type with HBV enrichment (**Supplementary Fig. S5b**).

To further delineate the HBV enrichment within hepatocytes, we re-clustered and re-integrated the hepatocytes of 11/24 patient samples that contained any HBV expression (**Fig. 4a**) and found that the only HBV-enriched subclusters were 0 and 3 (**Supplementary Table. S5**), with both subclusters enriched in 2/3 cohorts (**Fig. 4b**). We analyzed the CNV of each patient using inferCNV with the macrophages as reference cells and hepatocytes as observation cells (**Supplementary Fig. S6-8**); we noted generally the CNV clones with more HBV expression indeed mostly consisted of subclusters 0 and 3, while the clones with less HBV expression consisted of mainly subcluster 2. We picked three representative patients (from two cohorts) with well-defined CNV clones and a sufficient (>50) number of HBV-positive cells, P114_SRP318499, P725_SRP318499, P7_SRP278381, and generated an overall CNV for this set (**Fig. 4c**). The result shows that for patients in different cohorts, the clones with discernable, less ambiguous CNV patterns have a clear majority of cells with HBV expression compared to the other clones (**Fig. 4c**, green boxes).

We studied the CCI pattern of HBV-hepatocyte interactions and further grouped hepatocytes into HBV-positive (+) and normal (not HBV-positive) hepatocytes. CCIs in HBV-positive subclusters 0 and 3 have higher relative strength than normal ones shown in **Fig. 4d**. Proteinase-activated receptors (PARs) signaling pathway is tested to be the most significant signaling pathway (**Fig. 4d and e**) across the HBV-enriched hepatocytes. PARs as the thrombin receptors are involved in thrombin-induced cell migration across a collagen transmembrane barrier [43]. Midkine (MK) is a growth factor that is tested to be a crucial role in HCC. It is involved in inflammatory responses, acts as an anti-apoptotic factor, and blocks anoikic to promote metastasis [44].

Identification of *H. pylori* reads in gastric cancer

We also conducted a meta-analysis of the two gastric cancer (GC) cohorts that have publicly available droplet scRNA-seq sequencing data, SRP215370 (Zhang et al. [11]) and SRP261119 (Kim et al. [12]). The former includes early GC samples (which we only used the two confirmed *H. pylori*+ patients) and the latter contains GC patient samples, the majority of which were known to be *H. pylori+*. In total, we applied Vulture on the early GC or GC samples of 15 patients in the two cohorts, amounting to 125,845 after all QC filtering. We used the same approach as the above HCC case study for clustering and integration and labelled the cell types in line with Kim et al. (SRP261119) (**Fig. 5a**). Microbial enrichment detection by cohort showed *H. pylori* enrichment in endothelial cells, fibroblasts, and macrophages in SRP215370, and enrichment only in pit mucous cells in SRP261119 (**Fig. 5c** and **Supplementary Table. S6**). The difference is also present in the *H. pylori* virulence of two cohorts shown in **Fig. 3b**. CagA virulence gene is only detected in the Zhang et al. cohort where cagA-positive strains are the strongest risk factor of gastric cancer [45].

Discussion

In recent years, the rapidly growing single-cell study sources have become a gold mine for the re-investigation of host-microbial interactions. But neither traditional practices on scRNA-seq studies nor the bioinformatics methods developed to detect microbes are capable of scalable meta-analysis for the

public open data on the cloud. Alternatively, cloud computing has become an essential piece of equipment. Here, we present Vulture, a cloud-based scalable framework for calling microbial RNA on public scRNA-seq resources. Vulture was benchmarked on data originating from various tissues, generated with different scRNA-seq platforms, and deposited in different formats. It is tested to be highly scalable and cost-effective. Because it runs 200 analyses within a similar duration and low cost compared to a single task. Moreover, running a single Vulture analysis on the local environment is substantially faster than previous methods. The reason is that Vulture provides an easily customizable combined reference. It performs read mapping once on while others map read to the host and then align unmapped reads to the microbe references, among other intermediate steps. Vulture runs faster by getting rid of complex preprocessing on unmapped reads. The combined reference is 30% larger than the host genome but indexing from alignment tools can compensate for the increased reference size. Also, Vulture is user-friendly because it supports multiple platforms and multiple input formats. We demonstrated that Vulture can readily provide an effective solution for viral calling meta-analysis on large-scale public data.

We applied Vulture and scRNA-seq analysis to public COVID-19 BALF cells, HCC samples, and GC samples. The COVID-19 analysis revealed that Vulture can identify co-infections of unexpected pathogens. The HCC samples discovered a potential crucial relationship between CNV and intracellular HBV. All those cases indicate that Vulture is highly valuable to study unknown mechanisms and treatments by mining large-scale single-cell data.

However, there are several limitations of Vulture. A key hinder to Vulture meta-analysis is the permission of data. Serval atlas-level databases have strict access permission which makes it hard to run cloud-based analysis on a large scale. Also, large-scale meta-data cleansing for raw sequencing files, which is essential to run parallel meta-analysis appropriately is difficult because sequencing files are generated by different protocols. A prospective solution is to incorporate biomedical natural language processing (bioNLP) models and search engine technologies to subtract metadata for Vulture. Ultimately, Vulture automatically digs the gold mine of host-microbial interactions in big data.

In summary, Vulture is a cloud-based scalable framework for calling microbial RNA on public scRNA-seq resources. It is not only highly scalable, cost-effective on the cloud, and substantially outperformed previous methods in local environments. We anticipate that Vulture will play a crucial role in the attempt to understand the unrevealed genetics of pathogenic diseases as the community gradually contributes to the increasing scale of single-cell data for host-microbial interactions.

Availability of source code and requirements

Project name: Vulture

Project home page: https://github.com/holab-hku/Vulture

Project tutorial page: https://hiyin.github.io/vulture-user-tutorial

Operating system(s): For local version: Linux; For docker version: platform independent.

Programming language: Python, Nextflow, and Perl

Other requirements: For local version: R=4.0.5; DropletUtils >= v1.10.2; Docker = 20.10.21; STAR >= v2.7.9a (default) or cellranger >= 6.0.0 or Kallisto|bustools >= 0.25.1 or salmon|alevin >= v1.4.0. For cloud version: AWS batch.

License: MIT license

RRID： RRID:SCR_024720

Data Availability

The datasets supporting the conclusions of this article are available in the SRA Run Selector repository [46]. They can be searched under the following project accessions numbers: SRP250732 [35], SRP279746 [14], SRP136347 [8], SRP278381 [9], SRP318499 [10], SRP215370 [11], and SRP261119 [12]. All supporting data and materials are available in the *GigaScience* GigaDB database [47].

List of abbreviations

AWS: Amazon Web Services; BALF: bronchoalveolar lavage fluid; BAM: Binary Sequence Alignment Map; CCI: cell-cell interaction; CNV: Copy number variation; GC: gastric cancer; HBV: hepatitis B virus; HCC: hepatocellular carcinoma; hMPV: human metapneumovirus; HSV: Herpes simplex viruses; scRNA-seq: single-cell RNA sequencing; UMIs: unique molecular identifiers.

Consent for publication

Not applicable

Competing Interests

The authors declare that they have no competing interests.

Funding

This work was supported in part by the AIR@InnoHK programme of the Innovation and Technology Commission of Hong Kong. The funding source had no role in the study design; in the collection, analysis, and interpretation of data, in the writing of the manuscript, and in the decision to submit the manuscript for publication.

Authors' contributions

JWKH conceived and designed the study. JC, KHOY, and DY designed and implemented the computational pipeline and the cloud framework. HYHW contributed to the collection of data. XD contributes to the testing of prototypes. JC and KHOY performed the case study analysis and implemented the data analytics. All authors wrote, read, reviewed the manuscript, and approved the final version.

Acknowledgements

The authors thank all their colleagues, particularly at $D^2$4H and The University of Hong Kong for their support and intellectual engagement.

References

[1] M. Levrero and J. Zucman-Rossi, "Mechanisms of HBV-induced hepatocellular carcinoma," *J. Hepatol.*, vol. 64, no. 1, Supplement, pp. S84–S101, Apr. 2016, doi: 10.1016/j.jhep.2016.02.021.

[2] L. E. Wroblewski, R. M. Peek, and K. T. Wilson, "Helicobacter pylori and Gastric Cancer: Factors That Modulate Disease Risk," *Clin. Microbiol. Rev.*, vol. 23, no. 4, pp. 713–739, Oct. 2010, doi: 10.1128/CMR.00011-10.

[3] Y. Tian, L. N. Carpp, H. E. R. Miller, M. Zager, E. W. Newell, and R. Gottardo, "Single-cell immunology of SARS-CoV-2 infection," *Nat. Biotechnol.*, vol. 40, no. 1, Art. no. 1, Jan. 2022, doi: 10.1038/s41587-021-01131-y.

[4] N. Drayman, P. Patel, L. Vistain, and S. Tay, "HSV-1 single-cell analysis reveals the activation of anti-viral and developmental programs in distinct sub-populations," *eLife*, vol. 8, p. e46339, May 2019, doi: 10.7554/eLife.46339.

[5] M. Shnayder *et al.*, "Defining the Transcriptional Landscape during Cytomegalovirus Latency with Single-Cell RNA Sequencing," *mBio*, vol. 9, no. 2, pp. e00013-18, Mar. 2018, doi: 10.1128/mBio.00013-18.

[6] Y. Steuerman *et al.*, "Dissection of Influenza Infection In Vivo by Single-Cell RNA Sequencing," *Cell Syst.*, vol. 6, no. 6, pp. 679-691.e4, Jun. 2018, doi: 10.1016/j.cels.2018.05.008.

[7] F. Zanini *et al.*, "Virus-inclusive single-cell RNA sequencing reveals the molecular signature of progression to severe dengue," *Proc. Natl. Acad. Sci.*, vol. 115, no. 52, Dec. 2018, doi: 10.1073/pnas.1813819115.

[8] B. Losic *et al.*, "Intratumoral heterogeneity and clonal evolution in liver cancer," *Nat. Commun.*, vol. 11, no. 1, Art. no. 1, Jan. 2020, doi: 10.1038/s41467-019-14050-z.

[9] A. Sharma *et al.*, "Onco-fetal Reprogramming of Endothelial Cells Drives Immunosuppressive Macrophages in Hepatocellular Carcinoma," *Cell*, vol. 183, no. 2, pp. 377-394.e21, Oct. 2020, doi:

10.1016/j.cell.2020.08.040.

[10] D. W.-H. Ho *et al.*, “Single-cell RNA sequencing shows the immunosuppressive landscape and tumor heterogeneity of HBV-associated hepatocellular carcinoma,” *Nat. Commun.*, vol. 12, no. 1, Art. no. 1, Jun. 2021, doi: 10.1038/s41467-021-24010-1.

[11] P. Zhang *et al.*, “Dissecting the Single-Cell Transcriptome Network Underlying Gastric Premalignant Lesions and Early Gastric Cancer,” *Cell Rep.*, vol. 27, no. 6, pp. 1934-1947.e5, May 2019, doi: 10.1016/j.celrep.2019.04.052.

[12] J. Kim *et al.*, “Single-cell analysis of gastric pre-cancerous and cancer lesions reveals cell lineage diversity and intratumoral heterogeneity,” *Npj Precis. Oncol.*, vol. 6, no. 1, Art. no. 1, Jan. 2022, doi: 10.1038/s41698-022-00251-1.

[13] A. Regev *et al.*, “The Human Cell Atlas,” *eLife*, vol. 6, p. e27041, Dec. 2017, doi: 10.7554/eLife.27041.

[14] P. Bost *et al.*, “Host-Viral Infection Maps Reveal Signatures of Severe COVID-19 Patients,” *Cell*, vol. 181, no. 7, pp. 1475-1488.e12, Jun. 2020, doi: 10.1016/j.cell.2020.05.006.

[15] W. Zhang, X. Xu, Z. Fu, J. Chen, S. Chen, and Y. Tan, “PathogenTrack and Yeskit: tools for identifying intracellular pathogens from single-cell RNA-sequencing datasets as illustrated by application to COVID-19,” *Front. Med.*, vol. 16, no. 2, pp. 251–262, Apr. 2022, doi: 10.1007/s11684-021-0915-9.

[16] C. Y. Lee *et al.*, “Venus: An efficient virus infection detection and fusion site discovery method using single-cell and bulk RNA-seq data,” *PLOS Comput. Biol.*, vol. 18, no. 10, p. e1010636, Oct. 2022, doi: 10.1371/journal.pcbi.1010636.

[17] A. Yang, M. Troup, P. Lin, and J. W. Ho, “Falco: a quick and flexible single-cell RNA-seq processing framework on the cloud,” *Bioinformatics*, vol. 33, no. 5, pp. 767–769, 2017.

[18] B. Li *et al.*, “Cumulus provides cloud-based data analysis for large-scale single-cell and single-nucleus RNA-seq,” *Nat. Methods*, vol. 17, no. 8, pp. 793–798, 2020.

[19] T. M. Delorey *et al.*, “COVID-19 tissue atlases reveal SARS-CoV-2 pathology and cellular targets,” *Nature*, vol. 595, no. 7865, pp. 107–113, 2021.

[20] R. C. Edgar *et al.*, “Petabase-scale sequence alignment catalyses viral discovery,” *Nature*, pp. 1–6, 2022.

[21] D. Karolchik *et al.*, “The UCSC genome browser database,” *Nucleic Acids Res.*, vol. 31, no. 1, pp. 51–54, 2003.

[22] M. Stano, G. Beke, and L. Klucar, “viruSITE—integrated database for viral genomics,” *Database*, vol.

2016, 2016. doi: 10.1093/database/baw162/2742079

[23] H. Li, “Minimap2: pairwise alignment for nucleotide sequences,” *Bioinformatics*, vol. 34, no. 18, pp. 3094–3100, Sep. 2018, doi: 10.1093/bioinformatics/bty191.

[24] B. Kaminow, D. Yunusov, and A. Dobin, “STARsolo: accurate, fast and versatile mapping/quantification of single-cell and single-nucleus RNA-seq data,” *bioRxiv*, 2021. doi: https://doi.org/10.1101/2021.05.05.442755

[25] G. X. Y. Zheng *et al.*, “Massively parallel digital transcriptional profiling of single cells,” *Nat. Commun.*, vol. 8, no. 1, p. 14049, Jan. 2017, doi: 10.1038/ncomms14049.

[26] P. Melsted *et al.*, “Modular, efficient and constant-memory single-cell RNA-seq preprocessing,” *Nat. Biotechnol.*, vol. 39, no. 7, pp. 813–818, 2021.

[27] A. Srivastava, L. Malik, T. Smith, I. Sudbery, and R. Patro, “Alevin efficiently estimates accurate gene abundances from dscRNA-seq data,” *Genome Biol.*, vol. 20, no. 1, pp. 1–16, 2019.

[28] A. T. Lun, S. Riesenfeld, T. Andrews, T. Gomes, J. C. Marioni, and others, “EmptyDrops: distinguishing cells from empty droplets in droplet-based single-cell RNA sequencing data,” *Genome Biol.*, vol. 20, no. 1, pp. 1–9, 2019.

[29] F. A. Wolf, P. Angerer, and F. J. Theis, “SCANPY: large-scale single-cell gene expression data analysis,” *Genome Biol.*, vol. 19, no. 1, pp. 1–5, 2018.

[30] R. Satija, J. A. Farrell, D. Gennert, A. F. Schier, and A. Regev, “Spatial reconstruction of single-cell gene expression data,” *Nat. Biotechnol.*, vol. 33, no. 5, pp. 495–502, 2015.

[31] K. Polański, M. D. Young, Z. Miao, K. B. Meyer, S. A. Teichmann, and J.-E. Park, “BBKNN: fast batch alignment of single cell transcriptomes,” *Bioinformatics*, vol. 36, no. 3, pp. 964–965, Feb. 2020, doi: 10.1093/bioinformatics/btz625.

[32] I. Korsunsky *et al.*, “Fast, sensitive and accurate integration of single-cell data with Harmony,” *Nat. Methods*, vol. 16, no. 12, Art. no. 12, Dec. 2019, doi: 10.1038/s41592-019-0619-0.

[33] S. Jin *et al.*, “Inference and analysis of cell-cell communication using CellChat,” *Nat. Commun.*, vol. 12, no. 1, pp. 1–20, 2021.

[34] A. P. Patel *et al.*, “Single-cell RNA-seq highlights intratumoral heterogeneity in primary glioblastoma,” *Science*, vol. 344, no. 6190, pp. 1396–1401, 2014.

[35] M. Liao *et al.*, “Single-cell landscape of bronchoalveolar immune cells in patients with COVID-19,” *Nat.*

Med., vol. 26, no. 6, pp. 842–844, 2020.

[36] M. Mahler, P.-L. Meroni, M. Infantino, K. A. Buhler, and M. J. Fritzler, “Circulating Calprotectin as a Biomarker of COVID-19 Severity,” *Expert Rev. Clin. Immunol.*, vol. 17, no. 5, pp. 431–443, May 2021, doi: 10.1080/1744666X.2021.1905526.

[37] W. A. Turski, A. Wnorowski, G. N. Turski, C. A. Turski, and L. Turski, “AhR and IDO1 in pathogenesis of Covid-19 and the ‘Systemic AhR Activation Syndrome:’ a translational review and therapeutic perspectives,” *Restor. Neurol. Neurosci.*, vol. 38, no. 4, pp. 343–354, Sep. 2020, doi: 10.3233/RNN-201042.

[38] U. Raudvere *et al.*, “g:Profiler: a web server for functional enrichment analysis and conversions of gene lists (2019 update),” *Nucleic Acids Res.*, vol. 47, no. W1, pp. W191–W198, Jul. 2019, doi: 10.1093/nar/gkz369.

[39] F. Coperchini, L. Chiovato, L. Croce, F. Magri, and M. Rotondi, “The cytokine storm in COVID-19: An overview of the involvement of the chemokine/chemokine-receptor system,” *Cytokine Growth Factor Rev.*, vol. 53, pp. 25–32, Jun. 2020, doi: 10.1016/j.cytogfr.2020.05.003.

[40] R. L. Chua *et al.*, “COVID-19 severity correlates with airway epithelium–immune cell interactions identified by single-cell analysis,” *Nat. Biotechnol.*, vol. 38, no. 8, pp. 970–979, Aug. 2020, doi: 10.1038/s41587-020-0602-4.

[41] C. Bleilevens *et al.*, “Macrophage Migration Inhibitory Factor (MIF) Plasma Concentration in Critically Ill COVID-19 Patients: A Prospective Observational Study,” *Diagnostics*, vol. 11, no. 2, p. 332, Feb. 2021, doi: 10.3390/diagnostics11020332.

[42] J. L. Caniglia, S. Asuthkar, A. J. Tsung, M. R. Guda, and K. K. Velpula, “Immunopathology of galectin-3: an increasingly promising target in COVID-19,” *F1000Research*, vol. 9, p. 1078, Sep. 2020, doi: 10.12688/f1000research.25979.2.

[43] R. Kaufmann *et al.*, “Thrombin-mediated hepatocellular carcinoma cell migration: Cooperative action via proteinase-activated receptors 1 and 4,” *J. Cell. Physiol.*, vol. 211, no. 3, pp. 699–707, 2007, doi: 10.1002/jcp.21027.

[44] A. Gowhari Shabgah *et al.*, “Shedding more light on the role of Midkine in hepatocellular carcinoma: New perspectives on diagnosis and therapy,” *IUBMB Life*, vol. 73, no. 4, pp. 659–669, 2021, doi: 10.1002/iub.2458.

[45] M. HATAKEYAMA, “Structure and function of Helicobacter pylori CagA, the first-identified bacterial protein involved in human cancer,” *Proc. Jpn. Acad. Ser. B Phys. Biol. Sci.*, vol. 93, no. 4, pp. 196–219, Apr. 2017, doi: 10.2183/pjab.93.013.

[46] Sequence Read Archive (SRA) [Internet]. Bethesda (MD): National Library of Medicine (US), National Center for Biotechnology Information; 2009 - [cited 2023 Nov 08]. Available from: https://www.ncbi.nlm.nih.gov/sra/

[47] Chen J; Yin D; H Wong HY; Duan X; O Yu KH; K Ho JW. Supporting data for "Vulture: Cloud-enabled scalable mining of microbial reads in public scRNA-seq data" GigaScience Database 2023. http://dx.doi.org/10.5524/102473

Figures

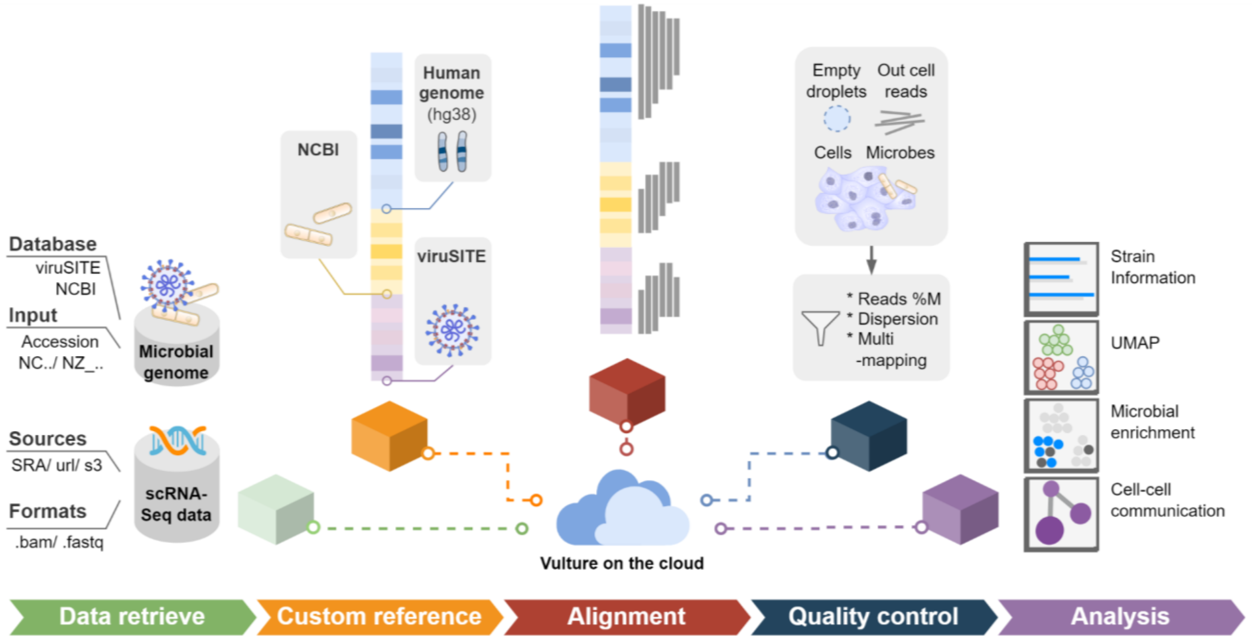

Fig. 1 Schematic diagram of Vulture for scRNA-seq microbial calling on the cloud. Vulture is a containerized computational framework composed of five procedures. The five steps include 1) multi-format scRNA-seq data and microbial genome retrieval; 2) custom combined reference construction; 3) reads alignment; 4) quality control, and 5) downstream analysis. All procedures in the Vulture architecture run as containerized applications on the Amazon Batch service.

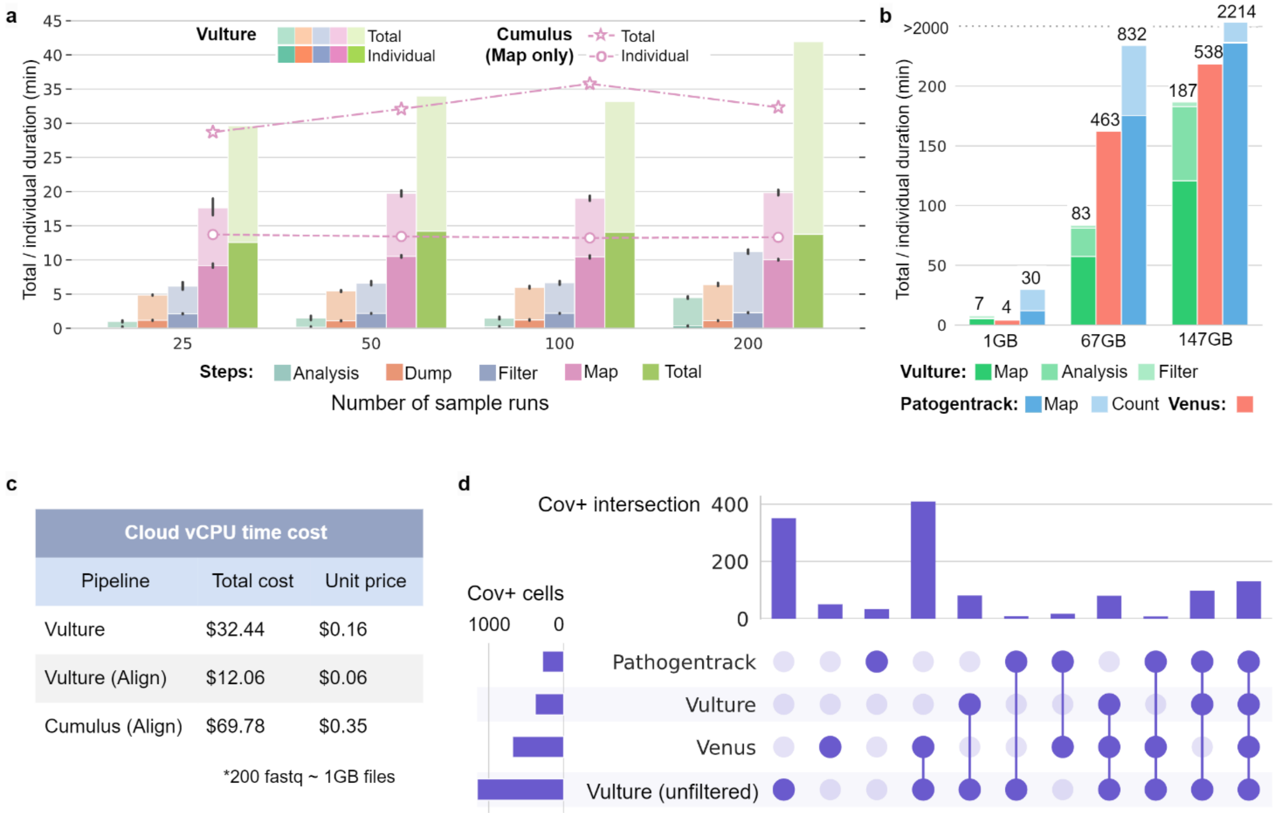

Fig. 2 Performance benchmark of the Vulture. **a)** Performances of the Vulture pipeline to run 25 to 200 parallel analyses. The performance is measured by execution duration (pastel), and vCPU time (saturated) with the respective number of parallel tasks. The time of each of the four steps in the Vulture pipeline is displayed separately. The line plot is the time for Cumulus to run 25 to 200 parallel read alignment tasks in comparison to the mapping step of Vulture. Total time (star-marked) and individual task durations (circle-marked) of different numbers parallel run processed by the two pipelines. **b)** Task duration comparison among viral calling methods to run one single analysis with different input file sizes. The local version of Vulture is composed of 3 steps (Map, Analysis, and Filter), Pathogentrack is composed of 2 steps (Map and Count) and Venues is end-to-end. The total duration of tasks is presented in stacked bar plots. **c)** Cost (in US dollars) of the computation resource needed to run 200 analyses on cloud platforms of Vulture and Cumulus. **d)** Consistency among viral calling methods. The consistency is estimated by measuring the intersections of virus-positive cells annotated by different tools. Vulture results and results before the filtering step are discussed separately.

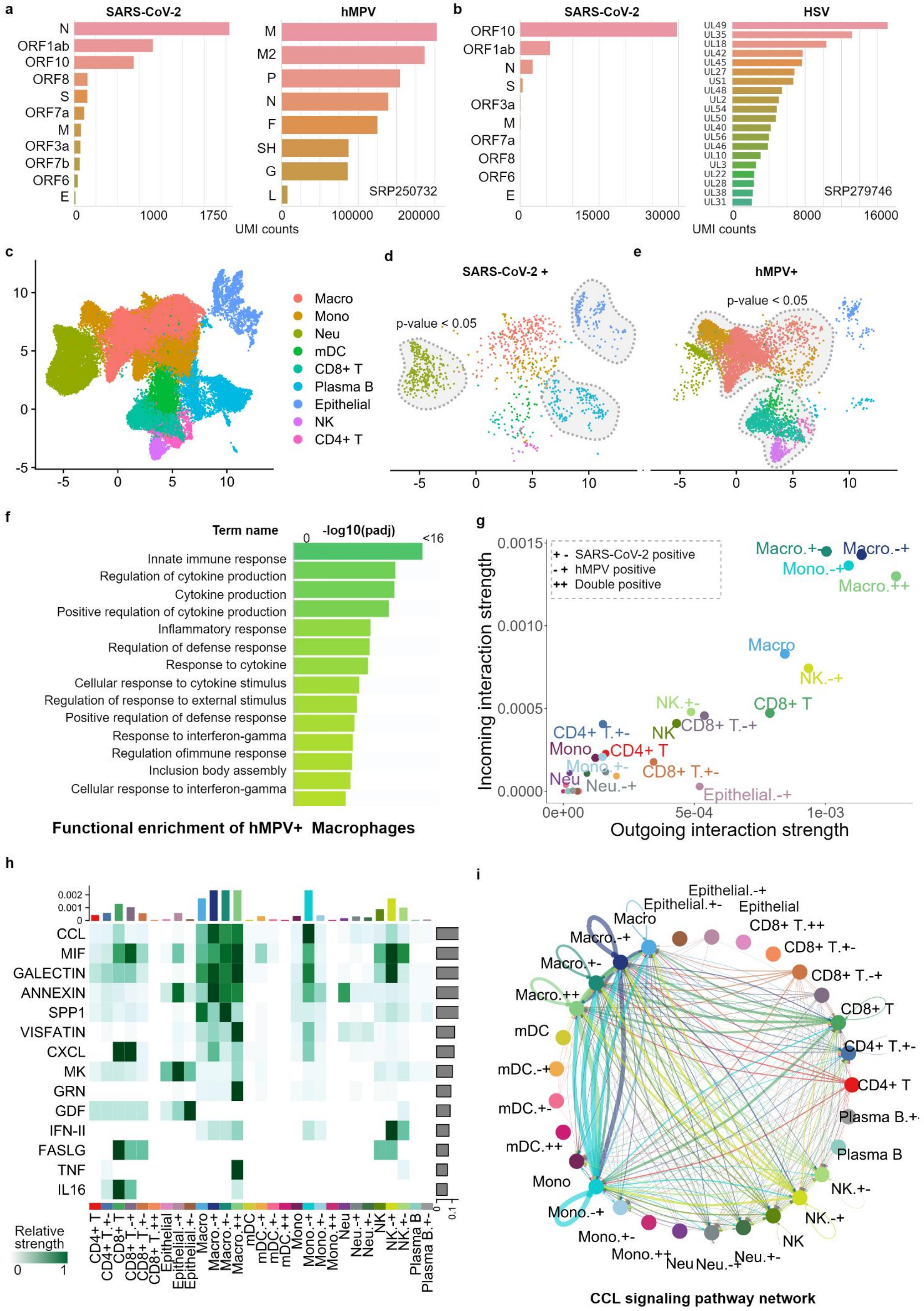
a
SARS-CoV-2
hMPV
SRP250732
UMI counts
b
SARS-CoV-2
HSV
SRP279746
UMI counts
c
Macro
Mono
Neu
mDC
CD8+ T
Plasma B
Epithelial
NK
CD4+ T
d
SARS-CoV-2 +
p-value < 0.05
e
hMPV+
p-value < 0.05
f
Term name
-log10(padj)
Innate immune response
Regulation of cytokine production
Cytokine production
Positive requlation of cytokine production
Inflammatory response
Requlation of defense response
Response to cytokine
Cellular response to cytokine stimulus
Regulation of response to external stimulus
Positive requlation of defense response
Response to interferon-gamma
Regulation ofimmune response
Inclusion body assembly
Cellular response to interferon-gamma
Functional enrichment of hMPV+ Macrophages
g
+ - SARS-CoV-2 positive
- + hMPV positive
++ Double positive
Incoming interaction strength
Outgoing interaction strength
h
CCL
MIF
GALECTIN
ANNEXIN
SPP1
VISFATIN
CXCL
MK
GRN
GDF
IFN-II
FASLG
TNF
IL16
Relative strength
i
CCL signaling pathway network

Fig. 3 Viral calling meta-analysis results on the COVID-19 BALF samples. **a)** and **b)** are transcript UMIs of three major detected viruses (SARS-CoV-2, hMPV, and HSV) from SRP250732 and SRP 279746, respectively. **c)** UMAP plot of the COVID-19 BALF data, cells are colored by cell type annotations. **d)** and **e)** are UMAP plots of the SARS-CoV-2 and hMPV infection, respectively. Infected cells are colored orange while other cells are gray. **f)** is the top 15 enriched Gene Ontology (GO) terms identified by functional enrichment analysis. **g)** cell-cell interaction (CCI) strengths across different cells grouped by cell types along with viral infections. **h)** presents enriched signaling pathways identified by CellChat. **i)** is the CCI among cell types through the CCL signaling pathway network.

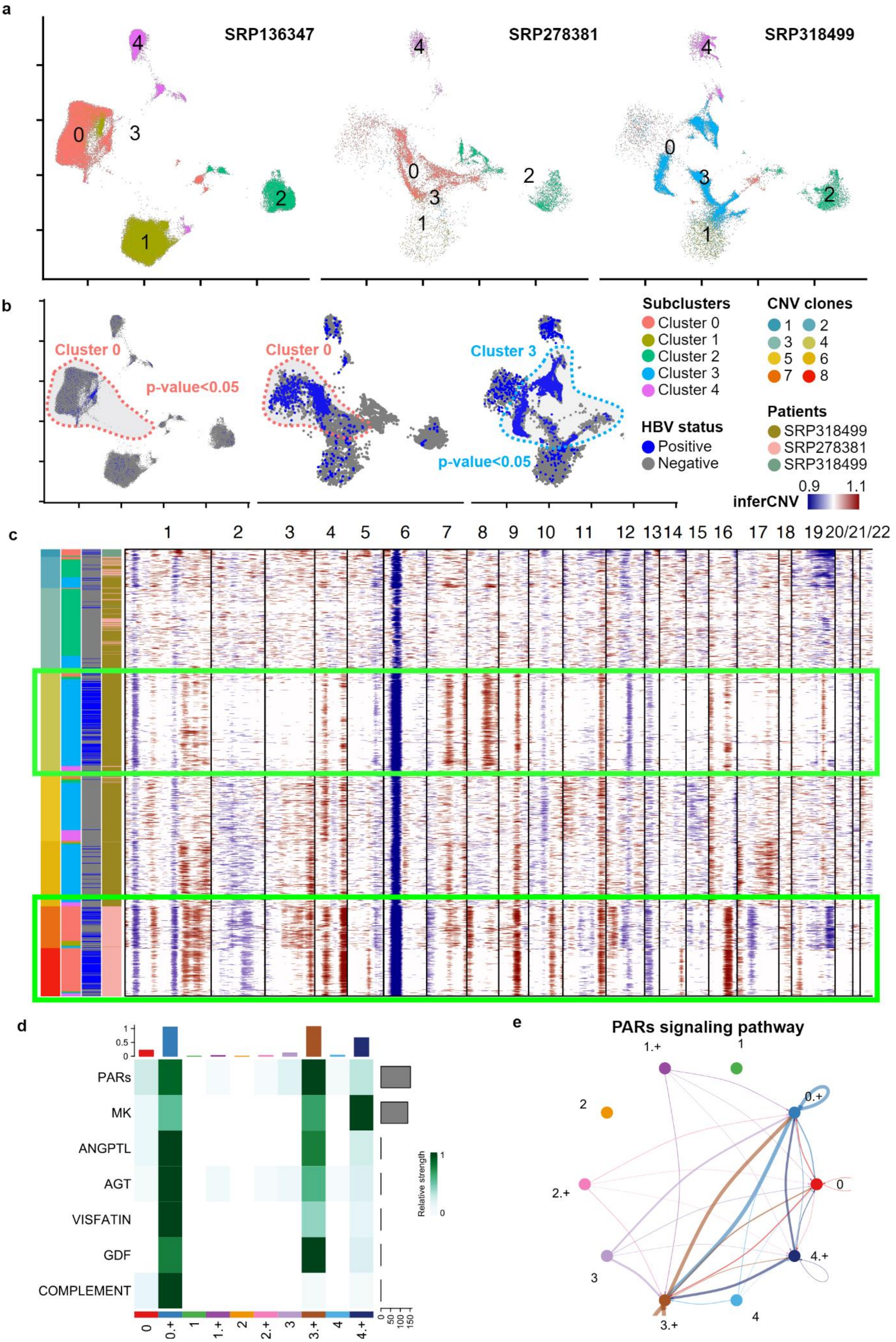
a
SRP136347
SRP278381
SRP318499
b
Cluster 0
p-value<0.05
Cluster 0
Cluster 3
p-value<0.05
Subclusters
Cluster 0
Cluster 1
Cluster 2
Cluster 3
Cluster 4
HBV status
Positive
Negative
CNV clones
Patients
SRP318499
SRP278381
SRP318499
inferCNV
c
d
PARs
MK
ANGPTL
AGT
VISFATIN
GDF
COMPLEMENT
Relative strength
e
PARs signaling pathway

Fig. 4 Viral calling meta-analysis results on the HCC hepatocytes. **a)** is a UMAP plot of the HBV-enriched hepatocytes, cells are colored by sub-clustering annotations. **b)** are UMAP plots of the intracellular HBV. HBV-positive cells are colored blue while other cells are gray. **c)** are the top 15 enriched Gene Ontology (GO) terms identified by functional enrichment analysis. **c)** heatmap of CNV inferred by inferCNV across chromosomes on the HCC hepatocytes. Cells are labeled by different annotations including CNV clones, sub-clusters, HBV status, and patients. **d)** presents enriched signaling pathways identified by CellChat of hepatocytes. **e)** is the CCI among cell types through the PARs signaling pathway network.

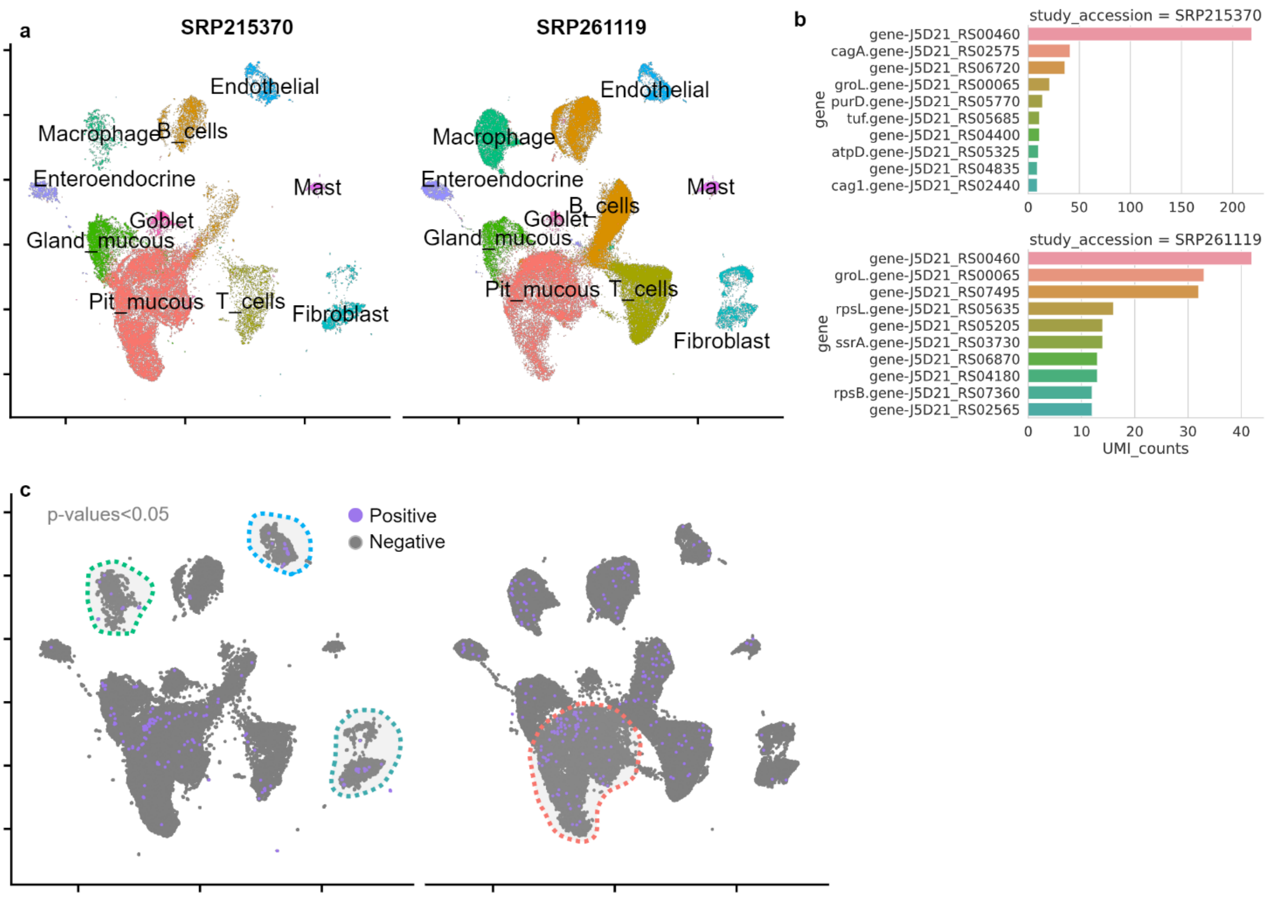

Fig. 5 Viral calling meta-analysis results on the GC sample. **a)** is a UMAP plot of the GC cells colored by cell type annotations. **b)** is the transcript UMIs of *H. pylori* identified in SRP215370 and SRP2161119, separately. **c)** are UMAP plots of the intracellular *H. pylori*. *H. pylori-positive* cells are colored purple while other cells are gray.

Tables

Data name	SRP accession	Disease	Tissue	#of runs	#of patients	Format
Liao et al. [35]	SRP250732	COVID-19	BALF	12	12	fastq
Bost et al. [14]	SRP279746	COVID-19	BALF	336	22	fastq
Losic et al. [8]	SRP136347	HCC	Tumor	7	2	bam
Sharma et al. [9]	SRP278381	HCC	Tumor	58	16	bam
Ho et al. [10]	SRP318499	HCC	Tumor	8	8	bam
Zhang et al. [11]	SRP215370	GC	Tumor	16	2	fastq
Kim et al. [12]	SRP261119	GC	Tumor	13	13	fastq

Table. 1 Overview of the datasets processed in this study.

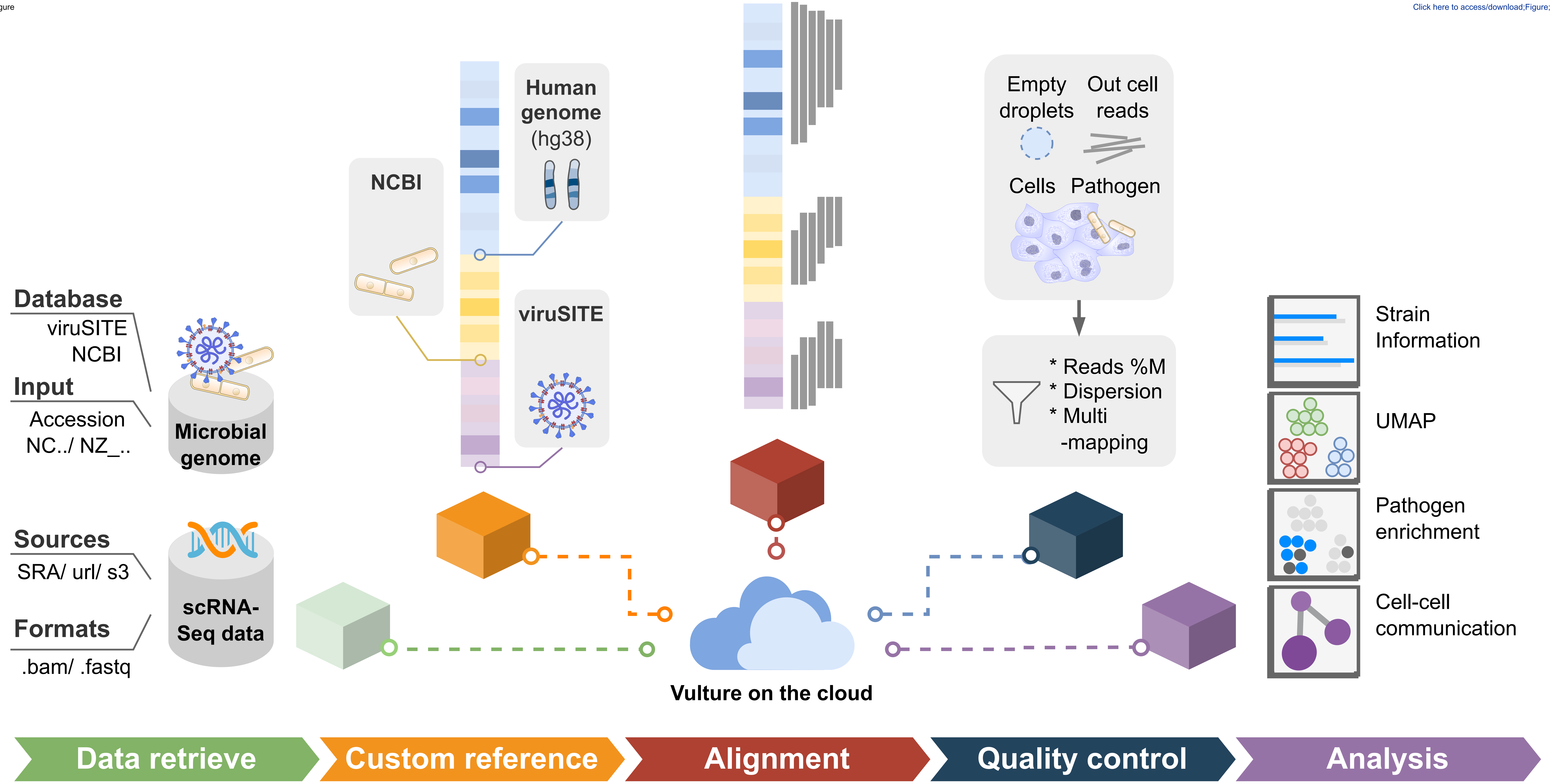
Database
viruSITE
NCBI
Input
Accession
NC../ NZ_..
Microbial genome
Sources
SRA/ url/ s3
Formats
.bam/ .fastq
scRNA-Seq data
NCBI
Human genome (hg38)
viruSITE
Empty droplets
Out cell reads
Cells
Pathogen
* Reads %M
* Dispersion
* Multi -mapping
Strain Information
UMAP
Pathogen enrichment
Cell-cell communication
Vulture on the cloud
Data retrieve
Custom reference
Alignment
Quality control
Analysis

Figure
Click here to access/download;Figure;Fig2.pdf

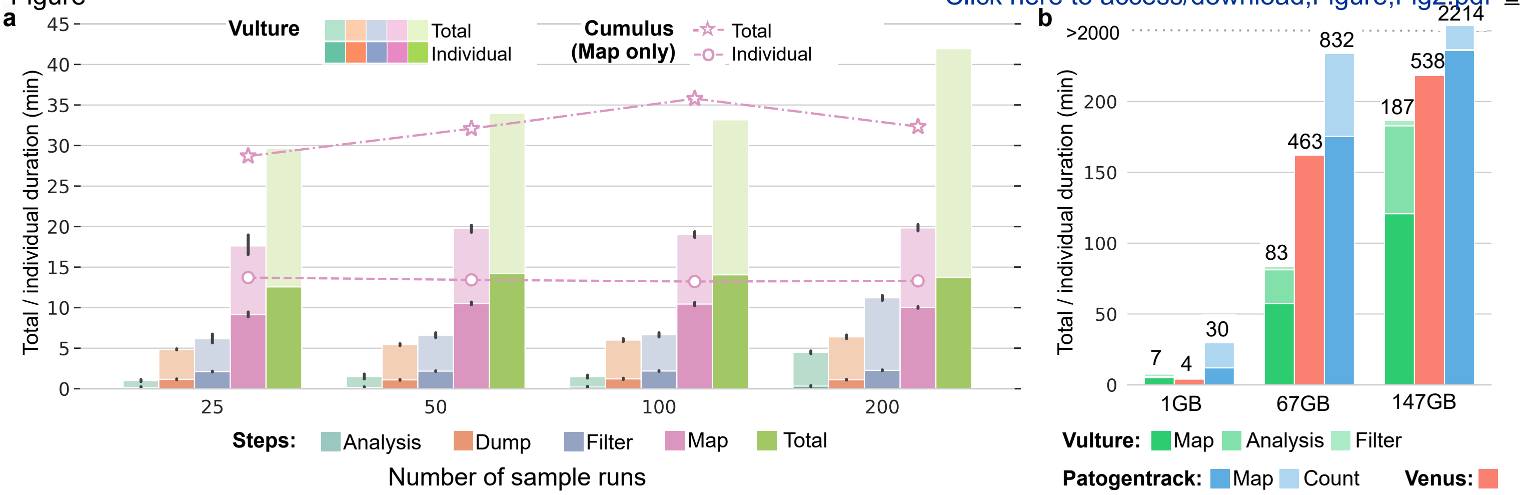

c

Cloud vCPU time cost		
Pipeline	Total cost	Unit price
Vulture	$32.44	$0.16
Vulture (Align)	$12.06	$0.06
Cumulus (Align)	$69.78	$0.35

*200 fastq ~ 1GB files

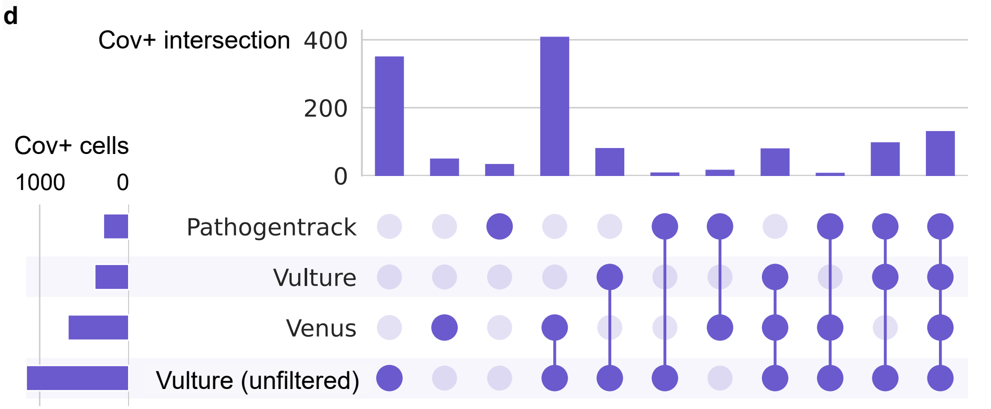

Figure

Click here to access/download;Figure;Fig3.pdf

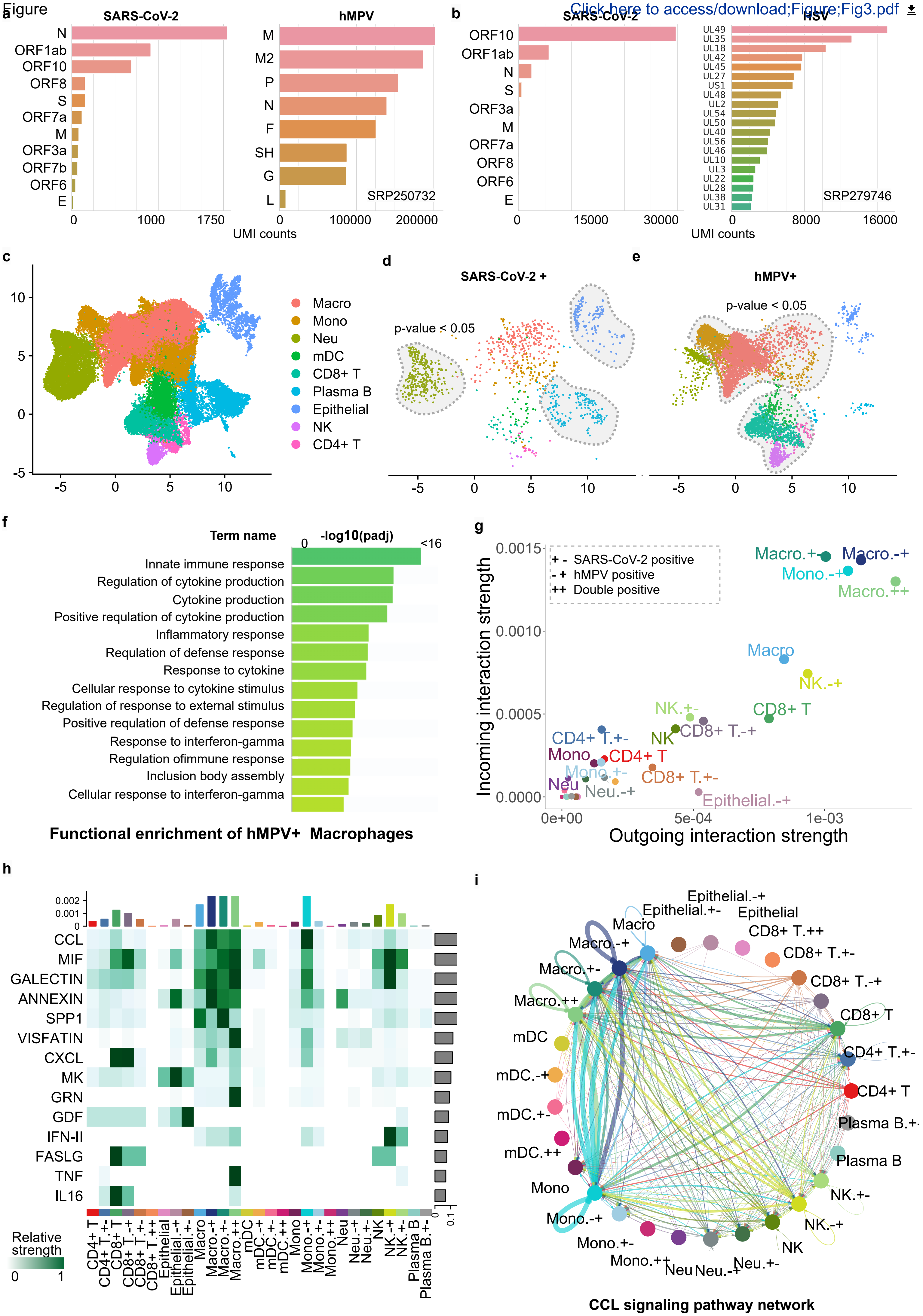

Figure

Click here to access/download;Figure;Fig4.pdf

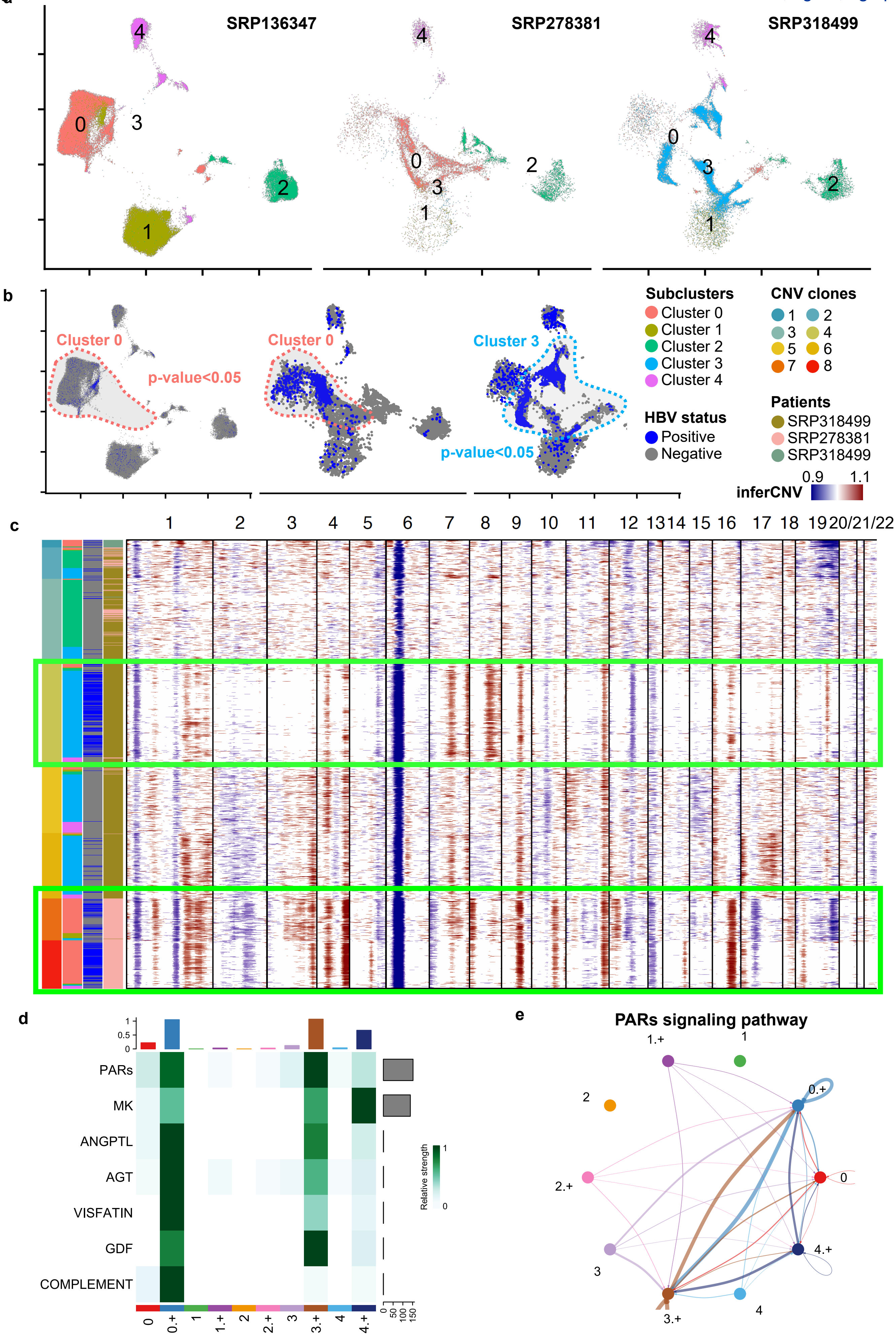

Figure

Click here to access/download;Figure;Fig5.pdf

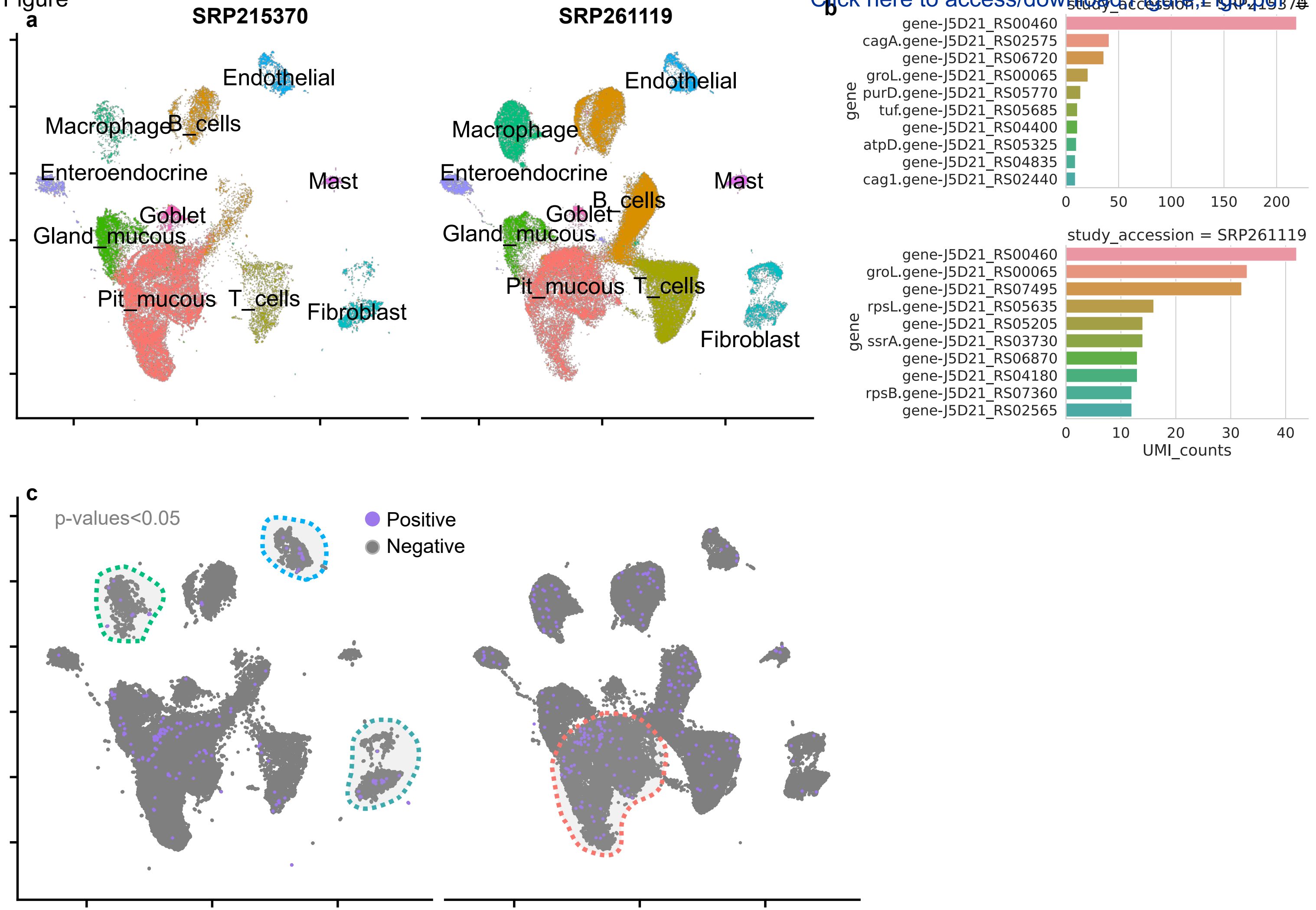

Click here to access/download
Supplementary Material
SupplementaryFile1.docx

Click here to access/download
Supplementary Material
SupplementrayTable1-6.xlsx

Click here to access/download
Supplementary Material
SupplementrayTable7.csv

Personal Cover Click here to access/download;Personal Cover;Cover_letter_revision.pdf

Dr Scott Edmunds
Editor-in-Chief
GigaScience

Dear Dr Edmunds,

Manuscript Revision Submission

We are writing to submit a revised manuscript entitled *"Vulture: Cloud-enabled scalable mining of microbial reads in public scRNA-seq data" (ID: GIGA-D-23-00124)* to be published as a research paper *in the journal GigaScience.* We greatly appreciate the insightful feedback from the reviewer, which has greatly contributed to the enhancement of our work.

Based on the feedback received, we have substantially revised the manuscript to address the reviewers' concerns and to improve the overall quality of the paper. In summary, we have

1. Performed a more systematic evaluation of Vulture's performance including measures such as step-by-step time consumption, as depicted in the revised **Fig. 2.**
2. The single-cell analysis for the Liao et al. cohort (**Fig. 3**), is re-performed by the Seurat pipeline to ensure the analysis consistency across cohorts, addressing the concern identified by the reviewers.
3. We have also significantly improved the documentation, particularly for the Vulture local installation, Docker usage, and an informative video tutorial that guides users through the installation and usage process.

Details of these and other minor changes are listed in the attached response letter. Furthermore, to address reviewer 1's concern about improving visibility of our work, we will promote our paper and source code through social media and other channels as widely as possible.

We hope that the revised manuscript can meet the high standards *of GigaScience*. We thank you and your editorial team's effort in facilitating the peer review process.

Yours sincerely,

Joshua W. K. Ho (on behalf of all authors)
