# Supplementary material for: Vulture: cloud-enabled scalable mining of microbial reads in public scRNA-seq data
Source: Gigascience. 2024 Jan 9;13:giad117. doi: 10.1093/gigascience/giad117 (PMC10776309; doi:10.1093/gigascience/giad117)
Supplement: giad117_Response_to_Reviewer_Comments_Original_Submission [file giad117_response_to_reviewer_comments_original_submission.pdf]

Author's Response To Reviewer Comments

Response letter to reviewer comments

Reviewer #1:
Comment 1: “The software, tested data and results are required to be uploaded on GitHub for peers to use, and conda and/or docker installation modes are recommended for software with complex dependencies. We will take software Star, Fork, and downloads of GitHub as one of the audience indicators … Now this project is only 2 stars. You need more people to take part in and interest in this project.”
Response 1: We have now released the Vulture Docker image on Docker hub (https://hub.docker.com/repository/docker/junyichen6/vulture/general) with a corresponding section “Running Vulture with docker image” (https://hiyin.github.io/vulture-user-tutorial/1.%20Running%20on%20local%20computer/4_Docker/) in the Vulture tutorial.
We are confident that all the changes in the revised version will improve the overall popularity of Vulture, especially after it is published.

Comment 2: “…. I found the GitHub links: https://github.com/holab-hku/Vulture. However, the readme.md show pipeline on AWS cloud. If I not have an AWS, how can I run it in my server….”
“Software installation and User tutorial are required in Readme.md or Wiki in GitHub. Please provide step by step protocol to deploy it in the laptop or server.”
“A video of software download, installation, operation, and result display is required with a computer or server without any related software installed, to make sure that any new user can perform the whole process according to the tutorial.”
Response 2: To further enhance the usability of Vulture applications beyond AWS, we have substantially updated the documentation to include step-by-step instructions to run Vulture on local servers (https://hiyin.github.io/vulture-user-tutorial/1.%20Running%20on%20local%20computer/1_Quickstart/).
We have created a comprehensive video tutorial in the page covering software download, installation, operation, and result display. Starting from scratch, users should be able to perform the entire process successfully. The combination of command line instruction, video tutorial, and docker instruction mentioned in Response 1, should enhance the usability of Vulture beyond AWS.

Comment 3: “The software is required to be posted on twitter and other social media, you can contact @ iMetaScience, @microbe_article etc. to get help in tweet or retweet. The number of Retweet, Like and View as one of the audience indicators.”
Response 3: We will of course actively promote Vulture on social media and other channels after the paper has completed the peer review process.

Comment 4: “Chinese is largest single langue science society. Provide the Chinese tutorial and video presentation of the software, contact meta-genome Official account for help to promote. The Number of readers, share and favorite also one of the audience indicators.”
Response 4: Adding Chinese language support is our plan for the next stage of development. In our experience, all Chinese users have no problem reading and using our software packages.

Comment 5: “According to the feedback from users in all over the world, the author continuously maintains and optimizes the method to ensure its availability, ease of use and advancement.”
Response 5: As seen by the effort made during this round of revision, of course we will make continuous efforts in maintaining the Vulture repository to ensure its availability, user-friendliness, and advancement.

Comment 6: “The software name should be unique, which is convenient to count the real users through all available resources (such as QIIME, ImageGP, and EasyAmplicon). However, the name vulture is unacceptable, due to million of hits in Google scholar.”
Response 6: There are indeed many widely-used bioinformatics tools with common names such as bowtie and cufflinks. These tools gained recognition within the bioinformatics community due to their performance, reliability, and wide adoption. ‘Vulture’ is a unique name for a bioinformatics tool and will be easily recognizable when it is widely used.

Comment 7: “The figures in your papers are diversity. However, I cannot find enough visualization function in your pipeline. The pipeline for integrated software is easy, the specific and diversity visualization plan is difficult. All the authors want their analysis result is ready-to-published.”
Response 7: The source code to support the generation of individual figures in this paper will be available on the GigaDB after being published. The functionalities provided by Vulture are already on par with other tools such as Viral-Track, PathogenTrack and Venus. I am sure most bioinformaticians can handle basic visualisation using the example source code we provide.

Comment 8: “Why only focus on the virus? Can this pipeline to generate all the microbiome, which is more interest and overview of the microbes.”
Response 8: Indeed Vulture can be used to identify any species in which a reference genome sequence can be provided. In the current version, Vulture’s database includes bacteria, archaea and viruses. To clarify this point, we have changed the title of this manuscript to ‘Vulture: Cloud-enabled scalable mining of microbial reads in public scRNA-seq data’.

Comment 9: “The figures need to be improved. Such as ImageGP (10.1002/imt2.5) can generate high quality figures and with reproducible scripts.”
Response 9: We have uploaded high-resolution figures in vectorized format. Additionally, we uploaded the figure reproduction scripts to the GIGA Science FTP server, which will be made available on GigaDB after the paper is published.

Comment 10:” Too many tables. Please move then as the supplementary table.”
Response 10: Tables. 2 to 7 are supplementary information to complement results presented in Fig. 3c, 4b, and 5c. We have made the necessary adjustments and moved these tables to Supplementary Table S1 to S6.

Comment 11: “The structure of the results appears not in concise. The authors should have presented their findings in around 3 mainly sections will be better. Some unimportant result in supplementary figures. In order to facilitate readers to better grasp the key points.”
Response 11: We have edited the result session, particularly the subsection “Vulture enables cloud-based discovery of Metapneumovirus reads in COVID-19 BALF samples”. The revised section describing Fig. 3 is more concise.

Reviewer #2:
Comment 12: In this study, Chen et al. introduce Vulture, a scalable cloud-based pipeline that performs microbial calling for single-cell RNA sequencing (scRNA-seq) data, enabling meta-analysis of host-microbial studies from the public domain. And they further applied Vulture to COVID-19, HCC, and gastric cancer human patient cohorts with public sequencing reads dataand discovered cell-type specific enrichment of SARS-CoV2, hepatitis B virus (HBV), and H. pylori positive cells. Generally speaking, this study is innovative, has good application potential, and can better assist the work of single cell research from the point of view of infection. I only a few minor questions that need the author to reply:

1. “Background: The first appearance of H. pylori should be replaced with its full name.”
Response 12: Fixed.

Comment 13. “Methods-Downstream analysis of scRNA-seq samples: Why use different tools (SCANPY/Seurat, BBKNN/Harmony) to analyze different datasets instead of using the same tool to analyze different datasets?”
Response 13: We initially employed different tools, such as SCANPY and Seurat, for analyzing distinct datasets due to scalability considerations. Given that the cohort of COVID-19 samples contains a larger number of cells compared to the HCC cohort, a Python-based pipeline was chosen for efficient analysis. In response to your question and to present more consistent results, we have revised our analysis. We now employ the Seurat/harmony pipeline for the downstream analysis and updated Fig. 3c, as well as

Supplementary Table S1, to enhance the coherence of findings across datasets.

Comment 14. “Cell-type enrichment of microbial UMI: format error of formula.”
Response 14: Fixed.

Comment 15. “Analyses-Page 11: "The statistical test identified that SARS-CoV-2 is enriched (p-value < 0.05) in epithelial cells, neutrophils, and plasma B cells (Fig. 3d and Table. 2)". It is best to highlight $p < 0.05$ data points in other colors rather than red squares. Why are there no $p < 0.05$ square in fig. 3e?”
Response 15: We have revised the color scheme in Fig. 3c, 3d, 4b, and 5c. Currently, virus/microbe-enriched cell types are highlighted by circles. The annotation for hMPV enrichment in Fig. 3d is added.

Comment 16. “Fig. 2a and 2b: There are 8 colors in figure 2a, however only 4 figure legend were showed. What do the four light-colored bar mean? And the same to Fig 2b.”
Response 16: We have revised Fig. 2 by merging Fig. 2 a, b to a single Fig. 2a to ensure that the comparison is accurately explained. The pastel-colored and saturated-colored bars in Fig. 2a represent the total time and the individual time of a computing process, separately. A legend to describe the color bar has been added to the figure. The total and individual time for the mapping process in Cumulus is demonstrated as line plots. The star-marked and round-marked dash lines in Fig. 2a represent the total time and the averaged individual time of the Cumulus marking time, separately.

Reviewer #3:
The authors aim to develop Cloud-enabled approaches for detecting viral reads in public single-cell RNA sequencing (scRNA-seq) data. This study makes a significant contribution to the identification of viruses and bacteria in public scRNA-seq data. Although the outcomes are satisfactory, the novelty of the proposed methods is limited. To date, no evidence has been provided to demonstrate their superiority over recently published methods (such as PathogenTrack and Venus, et al) when executed on a local machine. There are also several issues that need to be further addressed, as highlighted below:

Comment 17.” The documentation available on the GitHub pipeline does not explain how to utilize the latest virus database or how to incorporate a user's custom database. Because the virus database is updated very quickly now. It might be more appropriate if the author updates the database promptly or if one can customize and create their own database.”

Response 17: In fact, Vulture includes a dedicated function for constructing genomes. It downloads the latest list of microbes from both the ViruSITE and NCBI prokaryotes databases. These databases are frequently updated to ensure the inclusion of the most recent data. Our function is designed for users to maintain an up-to-date reference genome. We have also provided an enhanced step-by-step tutorial (https://hiyin.github.io/vulture-user-tutorial/3.%20Building%20your%20reference%20genome/1_Build/) to provide a more comprehensive demonstration of the genome construction process. Also, we will regularly upload the latest version of the reference genome to our repository to provide access to the most recent data for users.

Comment 18. “Figure 2a only has an overall comparison graph, it can be improved by adding detailed comparison graphs with Cumulus, PathogenTrack and Venus.”
“Figure 2b. The persuasiveness is not enough, it would be better to compare several pipeline platforms with similar functionalities or compare some specific steps, such as the four steps in figure 2a. By the way, all of these comparisons use comparison software developed by other same researchers, so please provide a detailed description of why the author's method is faster?”
Response 18: We have revised Fig. 2 by merging Fig. 2 a, b to a single figure mentioned in Response 15. The line plot in Fig. 2c has become a bar plot in Fig. 2b. It includes detailed individual steps duration of comparison among viral calling methods on one single analysis with different input file sizes. We have visualized 3 steps (Map, Analysis, and Filter) from Vulture, 2 steps (Map by Cellranger and Count by Pathogentrack) from Pathogentrack and Venues as a whole. The total duration of tasks is presented in stacked bar plots.

We have discussed the reason that makes Vulture run faster on local machines in the Discussion session. Vulture performs only one read mapping to the host-microbial reference genome, while others map reads to the host genome and then process a second round of read mapping to the microbe or virus references. For the cloud version, we discussed in the Result session that Vulture takes advantage of the AWS Batch spot capacity optimization technique. It ensured a higher availability of computational resource allocation, a larger number of concurrent tasks, and less response time for pending tasks compared to the unconfigurable Terra platform adopted by Cumulus.

Comment 19. “Figure 3c can be created with microbial clustering and non-microbial clustering to highlight the impact of virus identification on classification results.”
Response 19: We have revised the color scheme in Fig. 3c, 3d as described in the response to reviewer 2’s comment 2. Fig. 3 c, d and e are now spitted by sars-cov-2 positive and hMPV positive to highlight the impact of virus identification on classification results.

Comment 20. Fig. S1 It should be the "Quality control on read level".
Response 20: Fixed.
