# Supplementary material for: Vulture: cloud-enabled scalable mining of microbial reads in public scRNA-seq data
Source: Gigascience. 2024 Jan 9;13:giad117. doi: 10.1093/gigascience/giad117 (PMC10776309; doi:10.1093/gigascience/giad117)
Supplement: giad117_Reviewer_1_Report_Original_Submission — Yongxin Liu -- 6/20/2023 Reviewed [file giad117_reviewer_1_report_original_submission.pdf]

Reviewer Report

Title: Vulture: Cloud-enabled scalable mining of microbial reads in public scRNA-seq data

Version: Original Submission **Date:** 6/20/2023

Reviewer name: Yongxin Liu

Reviewer Comments to Author:

The manuscript presented by the authors provides a useful tool on the virome, which named "Vulture: Cloud-enabled scalable mining of viral reads in public scRNA-seq data", using a large and valuable dataset. The study is important in deepening our understanding of "virome in public data". However, there are some issues for improvement in the manuscript. Here are the requirements for new software that is good enough to be published:

Major comments:

1. The software, tested data and results are required to be uploaded on GitHub for peers to use, and conda and/or docker installation modes are recommended for software with complex dependencies. We will take software Star, Fork, and downloads of GitHub as one of the audience indicators. I found the GitHub links: https://github.com/holab-hku/Vulture. However, the readme.md show pipeline on AWS cloud. If I not have an AWS, how can I run it in my server. Now this project is only 2 stars. You need more people to take part in and interest in this project.
2. Software installation and User tutorial are required in Readme.md or Wiki in GitHub. Please provide step by step protocol to deploy it in the laptop or server.
3. A video of software download, installation, operation, and result display is required with a computer or server without any related software installed, to make sure that any new user can perform the whole process according to the tutorial.
4. The software is required to be posted on twitter and other social media, you can contact @ iMetaScience, @microbe_article etc. to get help in tweet or retweet. The number of Retweet, Like and View as one of the audience indicators.
5. Chinese is largest single langue science society. Provide the Chinese tutorial and video presentation of the software, contact meta-genome Official account for help to promote. The Number of readers, share and favorite also one of the audience indicators.
6. According to the feedback from users in all over the world, the author continuously maintains and optimizes the method to ensure its availability, ease of use and advancement.
7. The software name should be unique, which is convenient to count the real users through all available resources (such as QIIME, ImageGP, and EasyAmplicon). However, the name vulture is unacceptable, due to million of hits in Google scholar.
8. The figures in your papers are diversity. However, I cannot find enough visualization function in your pipeline. The pipeline for integrated software is easy, the specific and diversity visualization plan is difficult. All the authors want their analysis result is ready-to-published.
9. Why only focus on the virus? Can this pipeline to generated all the microbiome, which is more interest and overview of the microbes.

Minor comments:
1. The figures need to be improved. Such as ImageGP (10.1002/imt2.5) can generate high quality figures and with reproducible scripts.
2. Too many tables. Please move then as the supplementary table.
3. The structure of the results appears not in concise. The authors should have presented their findings in around 3 mainly sections will be better. Some unimportant result in supplementary figures. In order to facilitate readers to better grasp the key points.

Methods

Are the methods appropriate to the aims of the study, are they well described, and are necessary controls included? Choose an item.

Conclusions

Are the conclusions adequately supported by the data shown? Choose an item.

Reporting Standards

Does the manuscript adhere to the journal's guidelines on minimum standards of reporting? Choose an item.

Choose an item.

Statistics

Are you able to assess all statistics in the manuscript, including the appropriateness of statistical tests used? Choose an item.

Quality of Written English

Please indicate the quality of language in the manuscript: Choose an item.

Declaration of Competing Interests

Please complete a declaration of competing interests, considering the following questions:

- Have you in the past five years received reimbursements, fees, funding, or salary from an organisation that may in any way gain or lose financially from the publication of this manuscript, either now or in the future?
- Do you hold any stocks or shares in an organisation that may in any way gain or lose financially from the publication of this manuscript, either now or in the future?
- Do you hold or are you currently applying for any patents relating to the content of the manuscript?

- Have you received reimbursements, fees, funding, or salary from an organization that holds or has applied for patents relating to the content of the manuscript?
- Do you have any other financial competing interests?
- Do you have any non-financial competing interests in relation to this paper?

If you can answer no to all of the above, write 'I declare that I have no competing interests' below. If your reply is yes to any, please give details below.

I declare that I have no competing interests.

I agree to the open peer review policy of the journal. I understand that my name will be included on my report to the authors and, if the manuscript is accepted for publication, my named report including any attachments I upload will be posted on the website along with the authors' responses. I agree for my report to be made available under an Open Access Creative Commons CC-BY license (http://creativecommons.org/licenses/by/4.0/). I understand that any comments which I do not wish to be included in my named report can be included as confidential comments to the editors, which will not be published.

Choose an item.

To further support our reviewers, we have joined with Publons, where you can gain additional credit to further highlight your hard work (see: https://publons.com/journal/530/gigascience). On publication of this paper, your review will be automatically added to Publons, you can then choose whether or not to claim your Publons credit. I understand this statement.

Yes Choose an item.
