# Supplementary material for: Vulture: cloud-enabled scalable mining of microbial reads in public scRNA-seq data
Source: Gigascience. 2024 Jan 9;13:giad117. doi: 10.1093/gigascience/giad117 (PMC10776309; doi:10.1093/gigascience/giad117)
Supplement: giad117_Reviewer_1_Report_Revision_1 — Yongxin Liu -- 8/24/2023 Reviewed [file giad117_reviewer_1_report_revision_1.pdf]

Reviewer Report

Title: Vulture: Cloud-enabled scalable mining of microbial reads in public scRNA-seq data

Version: Revision 1 **Date:** 8/24/2023

Reviewer name: Yongxin Liu

Reviewer Comments to Author:

The manuscript presented by the authors provides a useful tool on the microbiome, which named "Vulture: Cloud-enabled scalable mining of microbial reads in public scRNA-seq data", using a large and valuable dataset. The study is important in deepening our understanding of "microbiome in public data". However, the author comments not fully address my concerned, there are some issues for improvement in the manuscript. Here are the requirements for new software that is good enough to be published:
1. A docker provided is better, however, most used install method conda is still missing.
2. The more microbial detect example is missing. Can you provide an example of using like Kraken2 full NCBI database (RefSeq) to check all the microbial is more useful.
3. Author still not promotion his software in social media. If no more people take part in use it, how can we know it's useful? The reviewers still have may work to do. Not have enough time to test this software. Just promote it in twitter and Chinese WeChat will help software better.
4. The software name should be unique, which is convenient to count the real users through all available resources (such as QIIME, ImageGP, and EasyAmplicon). However, the name vulture is unacceptable, due to millions of hits in Google scholar. Must be no hit is a unique nameï¼ŒOK? Otherwise, hardly to know the read number of users.
5. The source code to support the generation of individual figures in this paper will be available on the GigaDB after being published. Where to check by the reviewers?

Methods

Are the methods appropriate to the aims of the study, are they well described, and are necessary controls included? Choose an item.

Conclusions

Are the conclusions adequately supported by the data shown? Choose an item.

Reporting Standards

Does the manuscript adhere to the journal's guidelines on [minimum standards of reporting?]() Choose an item.

Choose an item.

Statistics

Are you able to assess all statistics in the manuscript, including the appropriateness of statistical tests used? Choose an item.

Quality of Written English

Please indicate the quality of language in the manuscript: Choose an item.

Declaration of Competing Interests

Please complete a declaration of competing interests, considering the following questions:

- Have you in the past five years received reimbursements, fees, funding, or salary from an organisation that may in any way gain or lose financially from the publication of this manuscript, either now or in the future?
- Do you hold any stocks or shares in an organisation that may in any way gain or lose financially from the publication of this manuscript, either now or in the future?
- Do you hold or are you currently applying for any patents relating to the content of the manuscript?
- Have you received reimbursements, fees, funding, or salary from an organization that holds or has applied for patents relating to the content of the manuscript?
- Do you have any other financial competing interests?
- Do you have any non-financial competing interests in relation to this paper?

If you can answer no to all of the above, write 'I declare that I have no competing interests' below. If your reply is yes to any, please give details below.

None.

I agree to the open peer review policy of the journal. I understand that my name will be included on my report to the authors and, if the manuscript is accepted for publication, my named report including any attachments I upload will be posted on the website along with the authors' responses. I agree for my report to be made available under an Open Access Creative Commons CC-BY license (http://creativecommons.org/licenses/by/4.0/). I understand that any comments which I do not wish to be included in my named report can be included as confidential comments to the editors, which will not be published.

Choose an item.

To further support our reviewers, we have joined with Publons, where you can gain additional credit to further highlight your hard work (see: https://publons.com/journal/530/gigascience). On publication of this paper, your review will be automatically added to Publons, you can then choose whether or not to claim your Publons credit. I understand this statement.

Yes Choose an item.
