# Supplementary material for: Vulture: cloud-enabled scalable mining of microbial reads in public scRNA-seq data
Source: Gigascience. 2024 Jan 9;13:giad117. doi: 10.1093/gigascience/giad117 (PMC10776309; doi:10.1093/gigascience/giad117)
Supplement: giad117_Reviewer_2_Report_Original_Submission — Jingzhe Jiang -- 7/8/2023 Reviewed [file giad117_reviewer_2_report_original_submission.pdf]

Reviewer Report

Title: Vulture: Cloud-enabled scalable mining of microbial reads in public scRNA-seq data

Version: Original Submission **Date:** 7/8/2023

Reviewer name: Jingzhe Jiang

Reviewer Comments to Author:

In this study, Chen et al. introduce Vulture, a scalable cloud-based pipeline that performs microbial calling for single-cell RNA sequencing (scRNA-seq) data, enabling meta-analysis of host-microbial studies from the public domain. And they further applied Vulture to COVID-19, HCC, and gastric cancer human patient cohorts with public sequencing reads dataand discovered cell-type specific enrichment of SARS-CoV2, hepatitis B virus (HBV), and H. pylori positive cells. Generally speaking, this study is innovative, has good application potential, and can better assist the work of single cell research from the point of view of infection. I only a few minor questions that need the author to reply:
1. Background: The first appearance of H. pylori should be replaced with its full name.
2. Methods-Downstream analysis of scRNA-seq samples: Why use different tools (SCANPY/Seurat, BBKNN/Harmony) to analyze different datasets instead of using the same tool to analyze different datasets?
3. Cell-type enrichment of microbial UMI: format error of formula.
4. Analyses-Page 11: "The statistical test identified that SARS-CoV-2 is enriched (p-value < 0.05) in epithelial cells, neutrophils, and plasma B cells (Fig. 3d and Table. 2)". It is best to highlight $p < 0.05$ data points in other colors rather than red squares. Why are there no $p < 0.05$ square in fig. 3e?
5. Fig. 2a and 2b: There are 8 colors in figure 2a, however only 4 figure legend were showed. What do the four light-colored bar mean? And the same to Fig 2b.

Methods

Are the methods appropriate to the aims of the study, are they well described, and are necessary controls included? Choose an item.

Conclusions

Are the conclusions adequately supported by the data shown? Choose an item.

Reporting Standards

Does the manuscript adhere to the journal's guidelines on [minimum standards of reporting?]() Choose an item.

Choose an item.

Statistics

Are you able to assess all statistics in the manuscript, including the appropriateness of statistical tests used? Choose an item.

Quality of Written English

Please indicate the quality of language in the manuscript: Choose an item.

Declaration of Competing Interests

Please complete a declaration of competing interests, considering the following questions:

- Have you in the past five years received reimbursements, fees, funding, or salary from an organisation that may in any way gain or lose financially from the publication of this manuscript, either now or in the future?
- Do you hold any stocks or shares in an organisation that may in any way gain or lose financially from the publication of this manuscript, either now or in the future?
- Do you hold or are you currently applying for any patents relating to the content of the manuscript?
- Have you received reimbursements, fees, funding, or salary from an organization that holds or has applied for patents relating to the content of the manuscript?
- Do you have any other financial competing interests?
- Do you have any non-financial competing interests in relation to this paper?

If you can answer no to all of the above, write 'I declare that I have no competing interests' below. If your reply is yes to any, please give details below.

I declare that I have no competing interests

I agree to the open peer review policy of the journal. I understand that my name will be included on my report to the authors and, if the manuscript is accepted for publication, my named report including any attachments I upload will be posted on the website along with the authors' responses. I agree for my report to be made available under an Open Access Creative Commons CC-BY license (http://creativecommons.org/licenses/by/4.0/). I understand that any comments which I do not wish to be included in my named report can be included as confidential comments to the editors, which will not be published.

Choose an item.

To further support our reviewers, we have joined with Publons, where you can gain additional credit to further highlight your hard work (see: https://publons.com/journal/530/gigascience). On publication of this paper, your review will be automatically added to Publons, you can then choose whether or not to claim your Publons credit. I understand this statement.

Yes Choose an item.
