# Supplementary material for: Vulture: cloud-enabled scalable mining of microbial reads in public scRNA-seq data
Source: Gigascience. 2024 Jan 9;13:giad117. doi: 10.1093/gigascience/giad117 (PMC10776309; doi:10.1093/gigascience/giad117)
Supplement: giad117_Reviewer_3_Report_Original_Submission — Liuyang Zhao -- 7/18/2023 Reviewed [file giad117_reviewer_3_report_original_submission.pdf]

Reviewer Report

Title: Vulture: Cloud-enabled scalable mining of microbial reads in public scRNA-seq data

Version: Original Submission **Date:** 7/18/2023

Reviewer name: Liuyang Zhao

Reviewer Comments to Author:

The authors aim to develop Cloud-enabled approaches for detecting viral reads in public single-cell RNA sequencing (scRNA-seq) data. This study makes a significant contribution to the identification of viruses and bacteria in public scRNA-seq data. Although the outcomes are satisfactory, the novelty of the proposed methods is limited. To date, no evidence has been provided to demonstrate their superiority over recently published methods (such as PathogenTrack and Venus, et al) when executed on a local machine. There are also several issues that need to be further addressed, as highlighted below:
1.The documentation available on the GitHub pipeline does not explain how to utilize the latest virus database or how to incorporate a user's custom database. Because the virus database is updated very quickly now. It might be more appropriate if the author updates the database promptly or if one can customize and create their own database.
2. Figure 2a only has an overall comparison graph, it can be improved by adding detailed comparison graphs with Cumulus, PathogenTrack and Venus.
3. Figure 2b. The persuasiveness is not enough, it would be better to compare several pipeline platforms with similar functionalities or compare some specific steps, such as the four steps in figure 2a. By the way, all of these comparisons use comparison software developed by other same researchers, so please provide a detailed description of why the author's method is faster?
4. Figure 3c can be created with microbial clustering and non-microbial clustering to highlight the impact of virus identification on classification results.
5. Fig. S1 It should be the "Quality control on read level".

Methods

Are the methods appropriate to the aims of the study, are they well described, and are necessary controls included? Choose an item.

Conclusions

Are the conclusions adequately supported by the data shown? Choose an item.

Reporting Standards

Does the manuscript adhere to the journal’s guidelines on minimum standards of reporting? Choose an item.

Choose an item.

Statistics

Are you able to assess all statistics in the manuscript, including the appropriateness of statistical tests used? Choose an item.

Quality of Written English

Please indicate the quality of language in the manuscript: Choose an item.

Declaration of Competing Interests

Please complete a declaration of competing interests, considering the following questions:

- Have you in the past five years received reimbursements, fees, funding, or salary from an organisation that may in any way gain or lose financially from the publication of this manuscript, either now or in the future?
- Do you hold any stocks or shares in an organisation that may in any way gain or lose financially from the publication of this manuscript, either now or in the future?
- Do you hold or are you currently applying for any patents relating to the content of the manuscript?
- Have you received reimbursements, fees, funding, or salary from an organization that holds or has applied for patents relating to the content of the manuscript?
- Do you have any other financial competing interests?
- Do you have any non-financial competing interests in relation to this paper?

If you can answer no to all of the above, write 'I declare that I have no competing interests' below. If your reply is yes to any, please give details below.

I declare that I have no competing interests.

I agree to the open peer review policy of the journal. I understand that my name will be included on my report to the authors and, if the manuscript is accepted for publication, my named report including any attachments I upload will be posted on the website along with the authors' responses. I agree for my report to be made available under an Open Access Creative Commons CC-BY license (http://creativecommons.org/licenses/by/4.0/). I understand that any comments which I do not wish to be included in my named report can be included as confidential comments to the editors, which will not be published.

Choose an item.

To further support our reviewers, we have joined with Publons, where you can gain additional credit to further highlight your hard work (see: https://publons.com/journal/530/gigascience). On publication of this paper, your review will be automatically added to Publons, you can then choose whether or not to claim your Publons credit. I understand this statement.

Yes Choose an item.
